# Genomic and functional insights into *Lactiplantibacillus plantarum* UTNGt3 from Ecuadorian Amazon *Chrysophyllum oliviforme*: a safe and promising probiotic

**DOI:** 10.3389/fmicb.2025.1634475

**Published:** 2025-09-10

**Authors:** Gabriela N. Tenea, Jazmin Hidalgo, Gratiela Gradisteanu Pircalabioru, Victor Cifuentes

**Affiliations:** ^1^Biofood and Nutraceutics Research and Development Group, Faculty of Engineering in Agricultural and Environmental Sciences, Universidad Técnica del Norte, Ibarra, Ecuador; ^2^Research Institute of the University of Bucharest—ICUB, University of Bucharest, Bucharest, Romania; ^3^eBio-hub Centre of Excellence in Bioengineering, National University of Science and Technology Politehnica Bucharest, Bucharest, Romania

**Keywords:** cell adhesion, probiotic marker genes, *Lactiplantibacillus plantarum*, bacteriocin gene clusters, Caco-2 cell adhesion

## Abstract

**Introduction:**

*Lactiplantibacillus plantarum* is a versatile lactic acid bacterium (LAB) recognized for its probiotic potential, with key traits such as adhesion to intestinal epithelial cells and tolerance to bile salts and gastric acid being essential for its efficacy. In this study, we isolated and characterized *L. plantarum* strain UTNGt3 from *Chrysophyllum oliviforme* (Caimitillo) fruits collected in the Ecuadorian Amazon.

**Methods:**

Whole-genome sequencing, gene annotation, and *in silico* analyses were performed to explore genomic architecture, identify probiotic gene markers (PGMs), and assess safety features, including bacteriocin gene clusters (BGCs). *In vitro* assays evaluating bile salt and acid tolerance, cell surface hydrophobicity, auto-aggregation, and adhesion to Caco-2 intestinal epithelial cells were conducted to characterize probiotic traits. Additionally, the biocompatibility of UTNGt3 external metabolites was assessed using the MTT (3-(4,5-dimethylthiazol-2-yl)-2,5-diphenyl tetrazolium bromide) assay and LDH (lactate dehydrogenase) release assay on intestinal cells.

**Results:**

UTNGt3 genome spans 3,569,352 bp with 43.95% GC content. EggNOG analysis showed enrichment in genes related to general function prediction (11.89%), carbohydrate metabolism (8.97%), and transcription (8.45%), with 25.92% annotated as hypothetical proteins. No acquired antibiotic resistance or virulence genes were detected. Genome mining revealed three BGCs, *plantaricin_N*, *enterolysin_A*, and *plantaricin_W-beta*, associated with antimicrobial functions. Diverse PGMs involved in stress tolerance, adhesion, and vitamin biosynthesis were also identified. Phenotypic assays confirmed strong acid and bile tolerance, high auto-aggregation, surface hydrophobicity, and superior adhesion to Caco-2 cells compared to *E. coli*. Biocompatibility assays confirmed over 85% cell viability and minimal membrane damage, supporting their safety.

**Conclusion:**

These findings establish UTNGt3 as a safe, multifunctional probiotic candidate with potential for functional food applications and future gut health studies.

## 1 Introduction

The search for novel probiotic candidates has increasingly turned toward non-traditional sources, particularly fruits, which offer unique and underexplored reservoirs of lactic acid bacteria (LAB) with specialized adaptive traits. Unlike dairy-associated LAB, fruit-derived strains are naturally adapted to fluctuating environmental stresses such as variable pH, osmotic pressure, and microbial competition, making them promising candidates for robust probiotic applications (Papadimitriou et al., 2016). Fruits also present an ecological niche rich in polyphenols and complex carbohydrates, selective pressures that may drive the evolution of LAB with enhanced antioxidant activity, carbohydrate metabolism, and antimicrobial properties (Benavides et al., 2016; Carpi et al., 2022). This ecological adaptation not only supports their survival and function in the gastrointestinal tract but also augments their utility in functional food development and natural preservation systems. Thus, exploring LAB isolated from fruits expands the probiotic landscape beyond conventional dairy strains and provides new avenues for health-promoting innovations.

*Lactiplantibacillus plantarum* is a highly adaptable LAB with a long-standing history of association with fermented foods and human health. Initially described in the early 20th century from plant-based fermentations, *L. plantarum* has since been isolated from diverse ecological niches, including vegetables, dairy products, meat, and the gastrointestinal tract of humans and animals (Mao et al., 2021; Gizachew and Engidawork, 2024). This ecological versatility is attributed to its extensive genomic repertoire, enabling the utilization of a wide range of carbohydrates, tolerance to variable pH and osmotic conditions, and production of diverse secondary metabolites. The species holds considerable economic and biotechnological value, supported by its “Food Grade Status” and Generally Recognized As Safe (GRAS) designation, which facilitate its inclusion in functional foods and probiotic formulations (Li et al., 2021). Besides, is known to secrete an array of bioactive compounds, including bacteriocins, exopolysaccharides, short-chain fatty acids, and antioxidant molecules, which contribute to pathogen inhibition, modulation of the gut microbiota, and regulation of host immune responses (Alizadeh Behbahani et al., 2019). Additionally, certain *L. plantarum* strains are recognized for their potential as natural food preservatives due to their production of antimicrobial peptides, such as bacteriocins, which can inhibit spoilage and pathogenic microorganisms when applied as crude extracts or purified peptides (Pupa et al., 2021). A key safety concern is antibiotic resistance, as some strains exhibit intrinsic resistance, which may play a role in restoring gut microbiota following antibiotic treatment (Efsa et al., 2022).

Despite the broad recognition of *L. plantarum* as beneficial, probiotic properties are strain-dependent, with significant variation in gastrointestinal survival, adhesion to intestinal epithelial cells, and bioactive production (Carpi et al., 2022). Consequently, comprehensive functional and genomic characterization is essential for identifying strains with targeted health-promoting effects. Given its historical use in traditional fermentations such as sauerkraut, kimchi, and sourdough, alongside its demonstrated metabolic adaptability, *L. plantarum* remains a key candidate for the development of next-generation probiotics and symbiotic formulations (Behera et al., 2018).

In previous studies, we explored the microbiota associated with various Amazonian fruits to identify LAB strains with antimicrobial and probiotic potential (Benavides et al., 2016). The ecological origin of these LAB strains is fundamental to their genomic and functional characteristics, as environmental pressures drive microbial adaptation through mechanisms such as horizontal gene transfer, metabolic specialization, and stress response modulation (Tenea and Ortega, 2021; Carpi et al., 2022). Comparative genomic analyses of *L. plantarum* strains from diverse fruit sources have demonstrated niche-specific adaptations, revealing gene variants linked to stress tolerance and metabolite production (Tenea, 2022).

Building upon this ecological-genomic framework, the present study focuses on *Chrysophyllum oliviforme*, commonly known as Caimitillo, a tropical fruit belonging to the *Sapotaceae* family and native to the Amazon rainforest (Tropical Plants Database, 2025). Traditionally valued not only for its nutritional content but also for its medicinal properties, including antioxidant and anti-inflammatory activities, *C. oliviforme* represents an untapped ecological niche for the isolation of beneficial LAB. Given its richly diverse and microbially competitive environment, this fruit offers a promising source for the discovery of *Lactiplantibacillus* strains with unique probiotic traits and potential functional applications.

In the present study, we report the isolation and genomic characterization of *L. plantarum* UTNGt3 from Caimitillo, highlighting the dynamic interplay between ecological pressures and genomic evolution. Whole-genome sequencing, comprehensive gene annotation, and functional analyses were performed to elucidate the strain genomic architecture and potential probiotic applications. *In silico* assessments confirmed the presence of probiotic gene markers and safety features, including annotated bacteriocin gene clusters. Additionally, key probiotic properties such as tolerance to bile salts and gastric acid, cell surface hydrophobicity, auto-aggregation capacity, and adhesion to Caco-2 intestinal epithelial cells were validated through *in vitro* assays. The biocompatibility of extracellular metabolites, specifically the cell-free supernatant (CFS), was further evaluated for cytotoxicity against Caco-2 cells. Thus, the connection between the fruit ecological background and the adaptive traits of UTNGt3 reinforces the significance of environmental origin in the functional potential of probiotic strains, supporting their use in food and biotechnology applications.

## 2. Materials and methods

### 2.1 Bacterial strain and growth conditions

The microorganism was isolated in October 2015 from caimitillo fruits (*Chrysophyllum oliviforme)*, collected from the Amazon rainforest in the Sucumbíos Province of Ecuador. Strain isolation was conducted using de Man, Rogosa, and Sharpe (MRS) agar medium. Samples were plated on MRS agar and incubated anaerobically at 37°C for 72 h. Distinct colonies were randomly selected and subjected to successive purification by re-streaking on MRS agar supplemented with 1% CaCO3 and 2% NaCl. Over 100 purified colonies per sample were evaluated based on standard phenotypic criteria, including Gram staining, motility, indole production, catalase activity, spore formation, and gas production from glucose. Antimicrobial activity was assessed against *Salmonella enterica* ATCC51741 and *Escherichia coli* ATCC25922. The isolate exhibiting the strongest inhibitory effect was selected for further analysis and designated as *L. plantarum* UTNGt3.

### 2.2 Genome sequencing

*De novo* whole-genome sequencing and assembly were carried out as previously outlined (Tenea et al., 2020) using a custom service from Macrogen Inc., (Seoul, Republic of Korea). The sequencing library was prepared by randomly fragmenting DNA and ligating 5’ and 3’ adapters. The quality of the raw sequence data was assessed using FastQC v0.11.5, and adapter sequences were removed with Trimmomatic v0.36. The filtered reads were further evaluated for various quality metrics, including total base count, read number, GC content, and other basic statistics. *De novo* assembly was performed using SPAdes 3.15.1 with multiple k-mer sizes, while k-mer analysis (using Jellyfish v2.2.10) provided information on genome coverage, heterozygosity, and estimated size. A De Bruijn graph assembler was employed for the final assembly, which resulted in 17,301,560 reads, a GC content of 43.42%, and a Q30 score of 93.11%. Genome validation was carried out through a mapping strategy and BUSCO analysis, yielding 37 contigs with a total length of 3,569,352 bp and an N50 value of 245,269 bp, indicating that at least half of the assembled genome is contained in contigs of this length or longer. Detailed assembly statistics and validation results are presented in Supplementary Tables 1–3.

### 2.3 Taxonomy and phylogenetic relationship

Species identification was performed using BLAST against the NCBI NT database, with ANI (Average Nucleotide Identity) values ≥95–96% confirming species identity (Richter and Rosselló-Móra, 2009). A circular genome map was generated using Proksee server (Grant et al., 2023). Additionally, for phylogenetic relationship single-copy orthologous genes were identified using OrthoMCL v2.0, a robust clustering algorithm for detecting orthologs and paralogs across multiple genomes (Li et al., 2003). The analysis was performed following the default parameters and recommended settings outlined in the OrthoMCL v2.0 user guide, ensuring accuracy and reproducibility. A total of 373 single-copy orthologous genes were retrieved, serving as essential genetic markers for phylogenetic analysis. The details of the bacterial strains used in this study are provided in Supplementary Table 4. The identified single-copy orthologous genes were aligned and concatenated into a supermatrix, which was then used to construct a maximum likelihood phylogenetic tree using RAxML 8.2.12 with the PROTGAMMAAUTO model, ensuring an optimal evolutionary inference (Stamatakis, 2014).

### 2.4 Gene prediction and functional annotation

The structural annotation of the UTNGt3 genome was performed using the PROKKA suite (Seemann, 2014). Coding sequences (CDS), rRNA, tRNA/tmRNA, signal leader peptides, and non-coding RNA were predicted using various tools: Prodigal (Hyatt et al., 2010) for CDS, RNAmmer for rRNA, Aragorn for tRNA/tmRNA, SignalP for signal peptides, and Infernal for non-coding RNA (Nawrocki and Eddy, 2013). Functional annotation was carried out using InterProScan (EBI InterPro) and the EggNOG database (Huerta-Cepas et al., 2019). Furthermore, the predicted genes were annotated using the Global Catalog of Microorganisms genome annotation pipeline^1^, referencing databases such as SwissProt, MetaCyc^2^ (which contains pathways involved in both primary and secondary metabolism) (Caspi et al., 2020), VFDB (a database of virulence factors) (Liu et al., 2019), PHI (Pathogen-Host Interaction database), KEGG (Kyoto Encyclopedia of Genes and Genomes), the Orthology Database of Prokaryotes, COG (Clusters of Orthologous Groups of proteins), and pFam (Mistry et al., 2021).

### 2.5 Prediction of antibiotic resistance genes, virulence factors, and pathogenicity

Comprehensive Antibiotic Resistance Database (CARD) (Jia et al., 2017) and the Resistance Gene Identifier (RGI) tool was used to detect the antibiotic resistance genes (Zankari et al., 2012). Additionally, acquired antimicrobial resistance genes and chromosomal mutations were identified via ResFinder 4.1 (Bortolaia et al., 2020) with a 90% identity threshold and a minimum sequence length of 60%. Putative virulence factors were predicted using the VFDB web server (Liu et al., 2019), while bacterial pathogenicity was determined using the PathogenFinder web server (Cosentino et al., 2013).

### 2.6 Prediction of biosynthetic gene clusters (BGCs)

Bacteriocin gene clusters (BGCs) related to antimicrobial compound production were identified using the BAGEL4 web server (de Jong et al., 2006). The BGC profile of UTNGt3 was then compared to that of the reference strain *L. plantarum* WCFS1 to assess similarities and differences in their antimicrobial potential.

### 2.7 *In vitro* probiotic and inhibitory characteristics

#### 2.7.1 Resistance to simulated gastric juice and bile salts

This assay was performed as previously described with some modifications (Alizadeh Behbahani et al., 2019). LAB cultures were anaerobically incubated at 37°C, then harvested by centrifugation (5000 × *g*, 5 min, 4°C) and washed twice with sterile Ringer’s solution (pH 7.2). The collected biomass was resuspended in synthetic gastric juice (pH 2.0) and incubated at 37°C for 4 h, with cell viability assessed hourly on MRS agar. The gastric juice composition included glucose (3.5 g/L), NaCl (2.05 g/L), KH2PO4 (0.60 g/L), CaCl2 (0.11 g/L), and KCl (0.37 g/L), adjusted to pH 2.0. After autoclaving at 121°C for 15 min, lysozyme (0.1 g/L), porcine bile (0.05 g/L), and pepsin (13.3 mg/L) were added. For bile salt tolerance, overnight LAB cultures (1 × 108 CFU/mL) were incubated in MRS broth containing 0.3% oxgall at 37°C for 4 h. Cell survival was calculated using the formula: Survival (%) = [(initial cell count – final cell count)/initial cell count] × 100. Results were compared with the reference strain *L. plantarum* ATCC8014 (LP).

#### 2.7.2 Assessment of hydrophobicity and autoaggregation

Hydrophobicity and autoaggregation were evaluated following established protocols (Liang et al., 2023). Bacterial suspensions were prepared at a concentration of 1 × 108 CFU/mL in phosphate-buffered saline (PBS, pH 7.2), and the initial absorbance (A0) was measured at 600 nm. For hydrophobicity assessment, equal volumes (1 mL) of hexane, ethyl acetate, and chloroform were added to the bacterial suspensions, followed by vortexing for 1 min and incubation for 1 h to allow phase separation. The absorbance of the aqueous phase (A1) was then recorded, and hydrophobicity (%) was calculated as: Hydrophobicity (%) = [(A0 - A1)/A0] × 100 (Wang et al., 2022). The results were compared against the reference strain *L. plantarum* (LP). For the autoaggregation assay, 4 mL of bacterial suspension was vortexed for 10 s, and the initial absorbance (A0) at 600 nm was recorded. The samples were then incubated at 37°C for 6, 8, and 24 h, and the absorbance (A_*t*_) was measured. Autoaggregation (%) = [1 - (At/A0)] × 100 was determined following the method described by Wang et al. (2022). The results were compared with both the LP reference strain.

#### 2.7.3 Adhesion to Caco-2 cells

The adhesion of UTNGt3 to Caco-2 cells was assessed as described by Wang et al. (2022) with slight modifications. Caco-2 (*in vitro* model for differentiated enterocytes) (HTB-37, ATCC) were cultured in RPMI 1640 medium (Thermo Fisher Scientific, #11875093) with 10% fetal bovine serum (FBS) + antibiotics (penicillin/streptomycin) at 37°C, 5% CO2, minimum 14–21 days for differentiation. The Caco-2 cells were cultured (2 × 10^5^ cells/mL) in 24-well tissue culture plates (Fisherbrand Surface Treated Sterile Tissue Culture Plates, # FB012929). Cells were grown until confluency (∼80–100%) and complete differentiation before probiotic testing. The Gt3 cells were inoculated (multiplicity of infection MOI = 10:1 or 100:1) onto a confluent layer of Caco-2 cells and incubated for 2 h at 37°C. The cells were washed twice with 500 μL PBS (1x) (pH = 7.2) to remove non-adherent bacteria. Then, the cells containing adherent bacteria were lysed with 500 μL of Triton-X (Biofroxx, Einhausen, Germany) solution (0.1% *v*/*v* in PBS). After incubating for 15 min, the solution containing released bacteria was taken, gradient-diluted, and plated on MRS agar. The adhesion rate was calculated following the equation adhesion ability (%) = (A_*x*_/A_*t*_) × 100, where *A*_*x*_ was the initial bacteria counts and *A*_*t*_ was the bacterial adhesion counts (Wang et al., 2022). The adherence rate was compared with the control *E. coli* ATCC11229 (non-probiotic bacteria). High adherence (> 20%) indicates increased probiotic potential. Moreover, to observe the Caco-2 cells adhesion of UTNGt3 and *E. coli* ATCC11229, Caco-2 cell was cultured (2 × 10^5^ cells/mL) in 24-well tissue culture plates containing coverslip. The suspension of bacterial cells was prepared as mentioned above and added to Caco-2 cells. After incubating for 2 h, each well was washed with PBS, then each well was incubated with methanol for 15 min to fix Caco-2 cells. After air-drying, the cells were stained using Giemsa staining. The coverslips were dried overnight. A biological microscope (Zeiss PrimoStar 3) was used to observe adherence under 100 × magnification.

### 2.8 Biocompatibility of external metabolites from UTNGt3

Caco-2 cells were used to evaluate the cytotoxic and viability effects of UTNGt3 metabolites using the MTT (3- (4,5-dimethylthiazol-2-yl)-2,5-diphenyltetrazolium bromide). Cell Proliferation Assay (Roche, #11465007001) and the LDH (Lactate dehydrogense) Cytotoxicity Analysis (Roche, # 11644793001). Caco-2 cells were seeded at a density of 1 × 10^6^ cells per well in 24-well flat-bottom tissue culture plates and cultured in RPMI 1640 medium (Thermo Fisher Scientific, # 11875093) supplemented with 10% fetal bovine serum (FBS) and 1% penicillin-streptomycin. The cells were allowed to adhere and reach sub-confluence over 24 h at 37°C in a humidified atmosphere with 5% CO2. *Lactobacillus* strains were grown overnight in MRS broth under appropriate anaerobic conditions until reaching approximately 108 CFU/mL. The cultures were diluted 1:100 in serum-free RPMI, resulting in a final concentration of approximately 1 × 106 CFU/well. The diluted cultures were centrifuged at 4000 × *g* for 3 min, and the resulting supernatants were used to treat Caco-2 cells. MRS medium alone served as a vehicle control, untreated cells were used as a negative control, and 1% Triton X-100 was used as a positive control for cytotoxicity (Zipperer, et al., 2023). After 24 h of treatment, LDH release into the culture medium was quantified as a measure of membrane damage. Fifty microliters of cell culture supernatant from each well were transferred to a new 96-well plate, mixed with 50 μL of LDH reaction mixture prepared according to the manufacturer instruction, and incubated for 30 min at room temperature in the dark. Absorbance was measured at 490 nm with a 620 nm reference filter using a microplate reader. Cytotoxicity was calculated as a percentage of maximal LDH release (from Triton X-100-treated cells) using the formula: Cytotoxicity (%) = [(Sample LDH – Low control)/(High control – Low control)] × 100. In parallel, the MTT assay was performed on a separate plate. After removing the treatment media, 10 μL of MTT labeling reagent was added to each well, followed by incubation for 4 h at 37°C. Then, 100 μL of solubilization solution was added to each well, and the plate was incubated overnight (or until complete dissolution of formazan crystals). Absorbance was measured at 570 nm with a reference wavelength between 630 and 690 nm. Cell viability was calculated relative to the untreated control using the formula: Cell viability (%) = [Absorbance (sample)/Absorbance (control)] × 100. All experiments were conducted in triplicate and repeated at least three times independently.

### 2.9 Statistical analysis

The results were expressed as means ± standard deviation (SD) from triplicate analyses. A one-way ANOVA was employed to assess cell viability under the different treatments with Tukey’s *post hoc* test applied for multiple comparisons. A significance level of *p* < 0.05 was applied (SPSS version 10.0.6, IBM, Armonk, NY, USA).

## 3. Results and discussion

### 3.1 Genomic analysis reveals unique features and adaptive potential of *L. plantarum* UTNGt3

**FIGURE 1:**
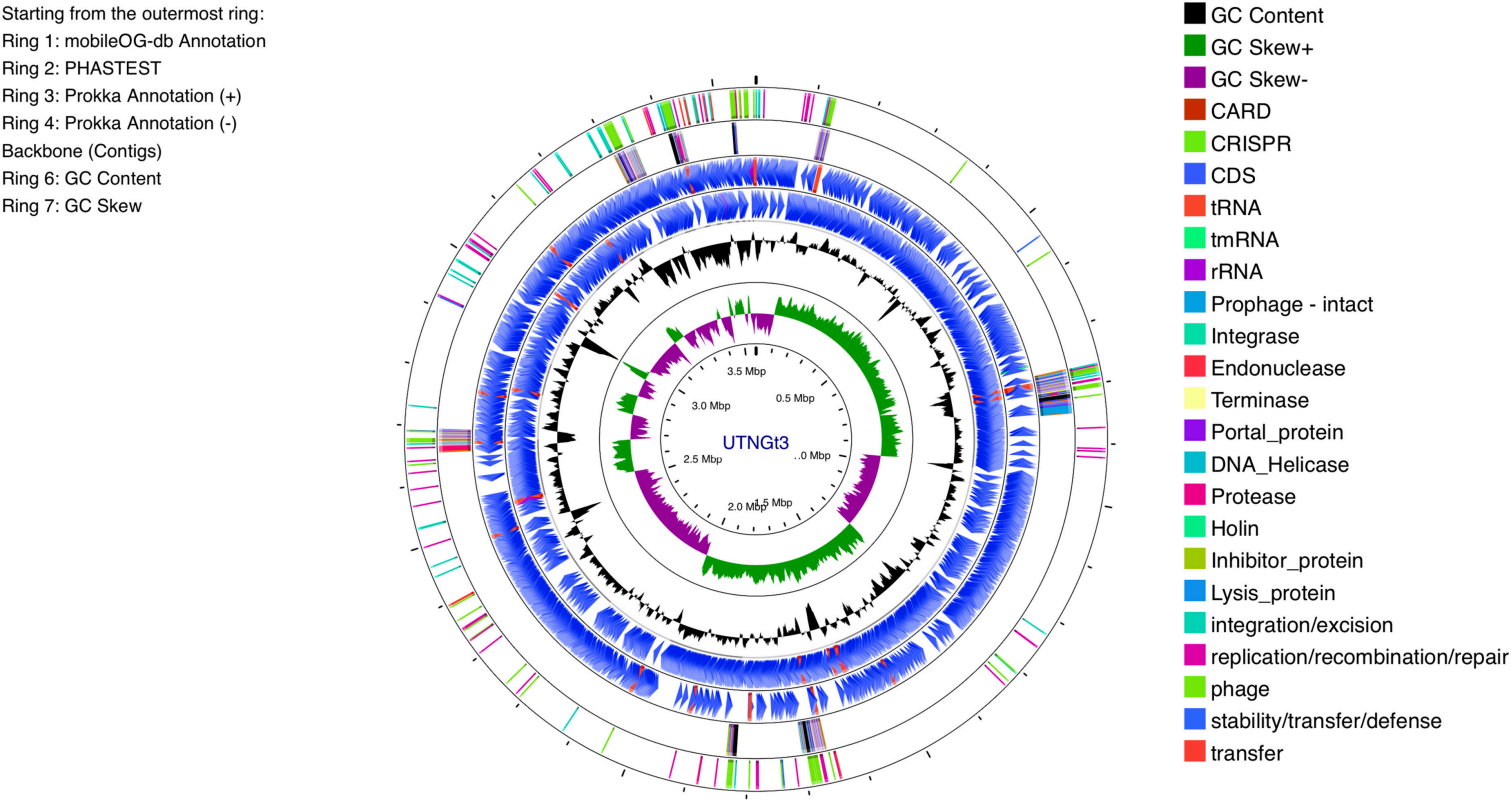
Circular genome diagram of *L. plantarum* UTNGt3 from the innermost circle to the outermost ring as follows: The first ring displays the MobileOG-db annotation; the second ring represents the prophages identified by PHASTEST; the third and fourth rings illustrate gene annotations with Prokka, showing the locations of CDSs, rRNA, tRNA, and tnRNA on the forward and reverse strands, respectively; the fifth ring highlights the backbone contigs; the sixth ring shows the GC content; and the seventh ring illustrates the GC skew (G + C/G-C).

The genomic characteristics of the UTNGt3 strain are summarized in Table 1. A comparative analysis with the previously sequenced genomes of *L. plantarum* UTNGt21A and *L. plantarum* UTNGt2 (Tenea and Ortega, 2021; Tenea and Ascanta, 2022), which were isolated from naranjilla and white cocoa fruits, respectively, as well as the reference human-origin strain *L. plantarum* WCFS1, is provided. The UTNGt3 strain possesses the largest genome (3,569,352 bp) and does not contain plasmids, a feature also observed in both UTNGt21A and UTNGt2 strains. The GC content of UTNGt3 (43.95%) is marginally lower than that of the other strains, particularly WCFS1 (45.6%). UTNGt3 encodes the highest number of total genes (3,538) and coding sequences (3,459), along with 83 tRNA genes, a value comparable to WCFS1 but exceeding that of UTNGt21A and UTNGt2. Notably, UTNGt3 harbors two CRISPR-Cas arrays, absent in UTNGt21A and WCFS1, but present in greater abundance in UTNGt2 (four), suggesting a potential role in adaptive immunity against foreign genetic elements. The genetic map of UTNGt3 is depicted in Figure 1. Additionally, UTNGt3 exhibits the presence of multiple prophage-associated elements, indicating past and potentially ongoing interactions with bacteriophages. Intact prophages, identified through PHASTEST annotation (Wishart et al., 2023), suggest that these elements may still retain functionality, influencing genome plasticity, adaptation, and ecological fitness. The presence of key phage-related genes, including integrases, portal proteins, terminases, DNA helicases, and lysis-associated proteins (holins, proteases, and inhibitors), highlights their role in potential phage integration, DNA packaging, and host cell lysis (Drulis-Kawa et al., 2015). These elements may contribute to horizontal gene transfer, enhancing genetic diversity and potentially impacting the strain probiotic properties, stress tolerance, or biofilm formation (Michaelis and Grohmann, 2023). Additionally, the coexistence of CRISPR elements suggests a dynamic interplay between phage defense mechanisms and prophage retention, which could regulate phage activation or suppression (Marchi et al., 2023). Overall, the prophage content of UTNGt3 underscores its genomic adaptability, potentially shaping its role as a probiotic strain, while also revealing the evolutionary forces that have influenced its genetic landscape. The gene annotation summary with different databases is shown in Table 2. The number of genes associated with COG (2362 genes) and KEGG (3239 genes) functional annotation categories are shown in Supplementary Figures 1A, B. Moreover, EggNOG analysis identified the most abundant gene category (11.89%) as associated with general functions, followed by 8.97% related to carbohydrate transport and metabolism, and 8.45% involved in transcription. Additionally, 25.92% of the genes encoding hypothetical proteins were classified as having an unknown function (Table 3). Genome annotation revealed that 3,221 proteins (3,180 Single EggNOG and 41 Multi EggNOG) matched entries in the EggNOG database, while no hits were found for 238 hypothetical proteins. These findings closely resemble those observed in the UTNGt21A strain (Tenea and Ascanta, 2022). In addition, the genome annotation revealed the presence of diverse CAZymes predicted by CAZy analysis, including multiple glycoside hydrolase (GH) families (Supplementary Table 5). The predominance of GH1, GH13, and GH25 suggests genomic potential for the degradation of a broad spectrum of carbohydrates such as starch, glycans, and peptidoglycans, which may contribute to nutrient acquisition and putative antimicrobial capabilities. Predicted glycosyltransferases, primarily from GT2 and GT4 families, indicate capacity for the biosynthesis of cell wall polysaccharides and exopolysaccharides, which are often associated with biofilm formation, host interactions, and probiotic-related traits. These genomic features collectively imply metabolic flexibility and ecological adaptability in both fermented food matrices and the gastrointestinal environment (Amin et al., 2021). KEGG pathway reconstruction further predicted complete metabolic routes for glycolysis, the tricarboxylic acid (TCA) cycle, and amino acid biosynthesis, supporting a potentially robust central metabolic network (Supplementary Figure 1). These genomic attributes suggest that UTNGt3 possesses enhanced genetic versatility, likely contributing to its ecological adaptation, genetic stability, and defense mechanisms in its native environment. However, these functions remain to be experimentally validated.

**TABLE 1:** Genome features of *L. plantarum* UTNGt3 compared with the references *L. plantarum* Gt21A, *L. plantarum* UTNGt2 and *L. plantarum* WCFS1strains.

Strain	*L. plantarum* UTNGt3	*L. plantarum* UTNGt21A	*L. plantarum* UTNGt2	*L. plantarum* WCFS1
Source	*Chrysophyllum oliviforme* (Caimitillo) fruit	*Solanum quitoense* (Lam.) wild fruit	*Theobroma grandifolium (cocoa)* fruit	Human saliva
Genome length (bp)	3,569,352	3,558,611	3,540,752	3,308,274
Plasmids	None	None	None	3
GC content (%)	43.95	43.96	44.53	45.6
Total number of genes	*3,538*	3524	3115	3174
Coding genes	*3,459*	3471	3052	3063
tRNA number of assembled genome	83	68	57	70
rRNA number of assembled genome	8	6	5	15
tmRNA number of assembled genome	1	1	1	3
CRISPR-Cas array*	2	0	4	0
Prophage (intact region)**	4	3	1	4
Antibiotic acquired genes***	None	None	None	None
Pathogenicity****	Non-human pathogen	Non-human pathogen	Non-human pathogen	Non-human pathogen

*CRISPRFinder (https://crisprcas.i2bc.paris-saclay.fr/CrisprCasFinder/Index);

**PHAge Search Tool Enhanced Release (PHASTER) (http://phaster.ca);

***ResFinder 4.1 (https://cge.cbs.dtu.dk/services/ResFinder/);

****PathogenFinder. (http://cge.cbs.dtu.dk/services/PathogenFinder/).

**TABLE 2:** Gene annotation summary.

Num of genes (#)	CARD	MetaCyc	PHI	CAZy	VFDB	SwissProt	KEGG	COG
3484	51 (1.46%)	443 (12.72%)	209 (6.00%)	143 (4.10%)	97 (2.78%)	1144 (32.84%)	3239 (92.97%)	2362 (67.80%)

CARD, Comprehensive Antibiotic Resistance Database; MetaCyc, database that contains pathways involved in both primary and secondary metabolism; PHI, The Pathogen-Host Interaction database is a biological database that contains curated information on genes experimentally proven to affect the outcome of pathogen-host interactions; CAZy, Carbohydrate-active enzyme; VFDB, virulence factor database; SwissProt; KEGG, Kyoto Encyclopedia of Genes and Genomes; COG, Clusters of Orthologous Groups of proteins.

**TABLE 3:** EggNOG category distribution of functional annotation results.

Eggnog	Description	Count	Ratio (%)
J	Translation, ribosomal structure and biogenesis	150	4.5970
A	RNA processing and modification	0	0.0000
K	Transcription	276	8.4585
L	Replication, recombination and repair	177	5.4245
B	Chromatin structure and dynamics	0	0.0000
D	Cell cycle control, cell division, chromosome partitioning	25	0.7662
Y	Nuclear structure	0	0.0000
V	Defense mechanisms	74	2.2679
T	Signal transduction mechanisms	78	2.3904
M	Cell wall/membrane/envelope biogenesis	181	5.5470
N	Cell motility	3	0.0919
Z	Cytoskeleton	0	0.0000
W	Extracellular structures	0	0.0000
U	Intracellular trafficking, secretion, and vesicular transport	25	0.7662
O	Posttranslational modification, protein turnover, chaperones	65	1.9920
C	Energy production and conversion	107	3.2792
G	Carbohydrate transport and metabolism	293	8.9795
E	Amino acid transport and metabolism	205	6.2826
F	Nucleotide transport and metabolism	85	2.6050
H	Coenzyme transport and metabolism	65	1.9920
I	Lipid transport and metabolism	61	1.8694
P	Inorganic ion transport and metabolism	140	4.2905
Q	Secondary metabolites biosynthesis, transport and catabolism	19	0.5823
R	General function prediction only	388	11.8909
S	Function unknown	846	25.9271
Total	–	3263	100

Count: number of genes; Ratio (%): % of genes.

The genome assembly data of UTNGt3 have been deposited in the NCBI Sequence Read Archive under BioProject ID PRJNA1116628^3^ and BioSample accession SAMN48548443, with the submission completed on May 17, 2025.

### 3.2 Phylogenomic analysis confirms UTNGt3 as a member of the *L. plantarum* lineage with high genetic similarity to reference probiotic strains

**FIGURE 2:**
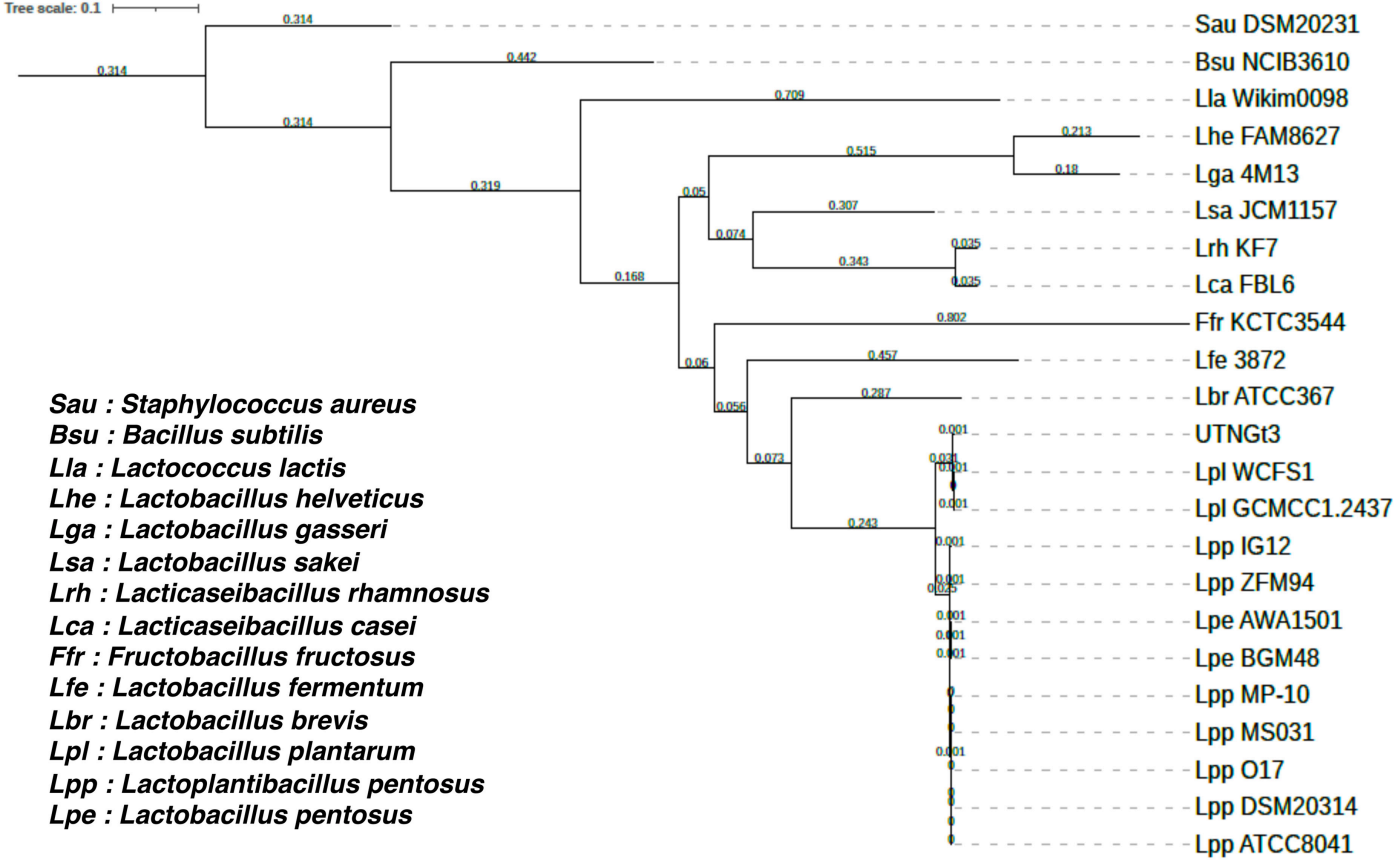
Phylogenetic tree of *Lactiplantibacillus plantarum* UTNGt3 and related bacterial strains based on single-copy orthologous genes. The tree was constructed using RAxML 8.2.12 with the PROTGAMMAAUTO model. Bootstrap support values are indicated at the nodes. UTNGt3 clusters within the *Lactiplantibacillus plantarum* clade, showing close evolutionary relationships with WCFS1 and GCMCC1.2437. The tree scale represents genetic distance.

The proportion based on the genus level as the result of the best hit (BLASTN analysis) for the entire contig was 100% matching *Lactobacillus* sp. The ANI values were 99.27% nucleotide identity and 83.81% alignment coverage with the genome of *Lactobacillus* D1501 (Supplementary Figure 2). The phylogenetic analysis reveals that UTNGt3 clusters closely with other *L. plantarum* strains, particularly WCFS1 and GCMCC1.2437, suggesting a high degree of genetic similarity and potential functional conservation (Figure 2). This positioning indicates that UTNGt3 likely shares key probiotic and antimicrobial traits characteristic of *L. plantarum* species. The strong bootstrap support values within its clade reinforce the robustness of this evolutionary relationship. Furthermore, the broader phylogeny shows that UTNGt3 belongs to a well-defined *Lactiplantibacillus* group, distinct from other *Lactobacillus* species such as *L. brevis* and *L. fermentum*. This classification supports its identity within the *L. plantarum* lineage and suggests that its genomic and metabolic characteristics align with those of established probiotic strains.

### 3.3 Genomic evidence of intrinsic resistance, stress adaptation, and non-pathogenicity

**FIGURE 3:**
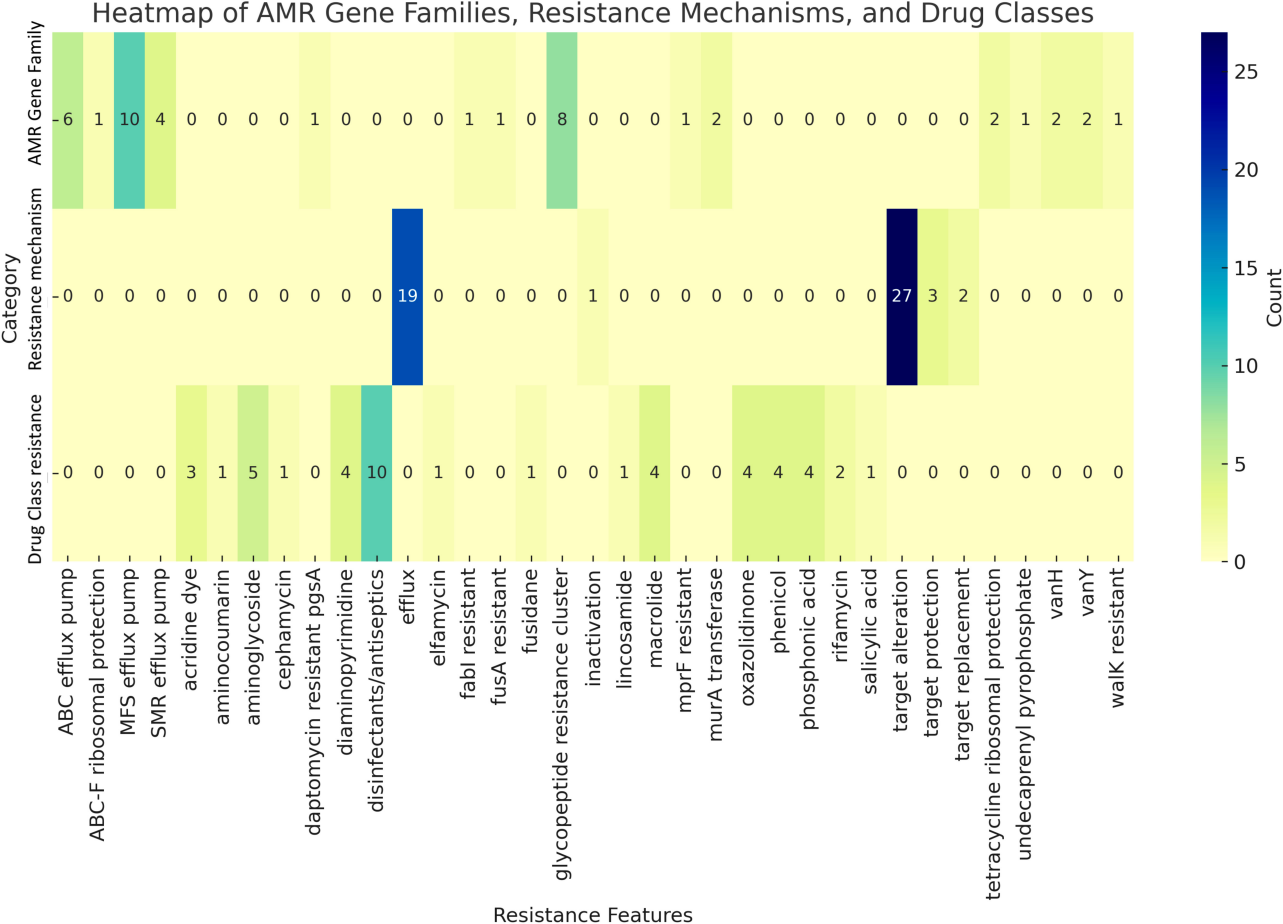
Antimicrobial markers prediction by category. The number of counts (genes) in shown.

Horizontal gene transfer enables microorganisms to acquire genetic material from coexisting organisms within their microenvironment, potentially enhancing their virulence, pathogenicity, or antimicrobial resistance (Jia et al., 2017). Genome analysis using CARD database identified the presence of two resistance-associated genes in the UTNGt3 strain: *vanY and vanH*, conferring resistance to glycopeptide antibiotics, and *qacJ*, which mediates resistance to disinfectant agents (Figure 3). These genes were not confirmed by Prokka and EggNOG annotation. Additionally, two tetracycline resistance genes, *tetA* (tetracycline-resistant protein class B) and *tetO* (COG0408), were annotated with EggNOG in the UTNGt3 genome. These genes are commonly found in *Lactiplantibacillus* species, including the reference strain WCFS1 (Kleerebezem et al., 2003). While intrinsic antibiotic resistance in lactobacilli is generally not considered a safety concern, the European Food Safety Authority (EFSA, 2018) panel emphasizes that strains intended for probiotic applications in humans or as feed additives in animals should not carry transferable antimicrobial resistance genes to mitigate potential risks of horizontal gene transfer. Furthermore, RGI analysis detected the *ClpL* gene in the UTNGt3 genome with 98.25% sequence identity, covering an alignment length of 2115 base pairs, suggesting a nearly complete match with known reference sequences. Located in contig gnl| MG| UTNGt3_4 between positions 17315 to 19429, this gene is associated with temperature resistance, indicating its potential role in heat stress adaptation. This trait may enhance the strain ability to survive under thermal fluctuations, which is particularly advantageous in probiotic applications or industrial fermentation processes. The annotation is supported by reference data (accession number CP023753), further reinforcing its relevance in bacterial stress response mechanisms. PathogenFinder analysis predicted UTNGt3 as a non-human pathogen with a 99.94% probability, indicating a highly confident classification. Seven genes, including *has*C (UTP-glucose-1-phosphate uridylyltransferase HasC), *clp*P (ATP-dependent Clp protease proteolytic subunit), *eno* (enolase), *tuf* (elongation factor Tu), *lis*R (two-component response regulator), *rfb*B (dTDP-glucose 4,6-dehydratase), and *rfb*A (glucose-1-phosphate thymidylyltransferase RfbA) were predicted as putative virulence factors with < 70% accuracy using the VFDB database with strict criteria (>80% identity and >60% coverage) (Supplementary Table 6). However, Prokka annotation confirmed the presence of enolase, ATP-dependent Clp protease proteolytic subunit and elongation factor Tu. In *Lactobacillus*, the ATP-dependent Clp protease proteolytic subunit (ClpP) is vital for protein quality control, ensuring cellular homeostasis by degrading damaged, misfolded, and short-lived proteins. This function is essential for the bacterium stress response and overall cellular maintenance (Queraltó et al., 2023). Although these genes were previously identified in the *Lactobacillus* probiotic genome, there is insufficient evidence to confirm their virulence. Additionally, other genes may contribute to various metabolic pathways, such as complex polysaccharide and glycoprotein synthesis (*has*C), as well as protein quality control and degradation (Murphy et al., 2023). The *clp*P gene, for instance, is involved in the ATP-dependent degradation of damaged or misfolded proteins, facilitating proteolysis within a barrel-shaped protease complex (Suokko et al., 2005). A BLASTP protein sequence homology search and alignment analysis revealed 100% identity between the queried protein and the membrane protein insertase YidC from multispecies within the *Lactiplantibacillus* genus. YidC contains a conserved YidC/Oxa1 C-terminal domain, a conserved region in the YidC/Oxa1/Alb3 family, which plays a crucial role in membrane protein insertion and assembly (Shanmugam and Dalbey, 2019). This domain is universally conserved among bacteria, facilitating the integration of nascent polypeptides into the cytoplasmic membrane, though it is notably absent in Archaea (Shanmugam and Dalbey, 2019). In addition, the enolase gene presence was confirmed by EggNOG DB annotation. Enolase in *Lactobacillus* is a multifunctional enzyme primarily involved in glycolysis, catalyzing the conversion of 2-phosphoglycerate to phosphoenolpyruvate (Castaldo et al., 2009). Besides enolase protein is related with the resistance to acidic and bile stress conditions, a key factor for probiotic capacity (Carpi et al., 2022). Beyond its metabolic role, enolase also functions as a “moonlighting protein” when localized on the bacterial surface, where it binds to host plasminogen, potentially facilitating adhesion, colonization, and immune modulation (O’Kelly et al., 2024). Additionally, its expression may be upregulated in response to environmental stress, contributing to bacterial survival in acidic conditions. These diverse functions highlight enolase importance not only in energy metabolism but also in *Lactobacillus* adaptation, host interactions, and probiotic activity (Castaldo et al., 2009).

### 3.4 Distinct bacteriocin gene clusters in UTNGt3 suggest enhanced antimicrobial potential and ecological competitiveness

In *Lactobacillus*, the BGCs are structured into distinct functional modules that govern bacteriocin synthesis, regulation, secretion, and immunity, as evidenced by the presence of predicted promoters, terminators, and various associated genes. The size, composition, and arrangement of these clusters differ significantly among *L. plantarum* strains (Zhang et al., 2022). Recent studies have shown that *Lactobacillus* strains isolated from diverse environments produce compounds with antimicrobial activity against several food bacteriocins (Fernandes and Jobby, 2022).

**FIGURE 4:**
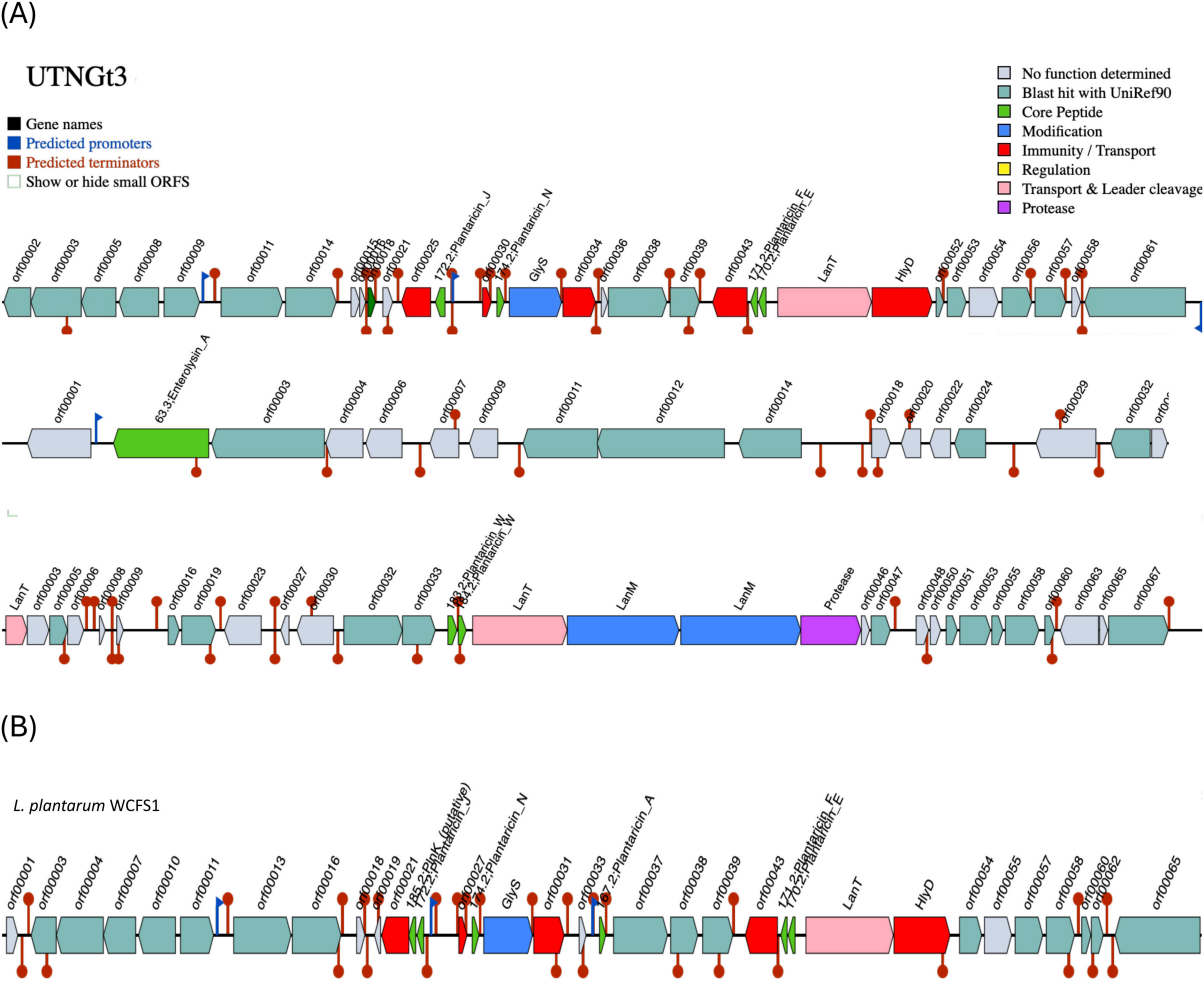
Bacteriocin cluster genes organization. Area of Interest (AOI) of *L. plantarum* UTNGt3 **(A)** and *L. plantarum* WCFS1 **(B)**. Legend: red blocks: immunity and transport; green arrow: core peptide; pink block: transport and leader cleavage; blue block: peptide modifications; grey blocks: no function determined.

The BGCs prediction analysis identified three AOI regions within the UTNGt3 genome (Figure 4A) being located on contig 1.21 (positions 441863–469378) and classified as *plantaricin_N*, contig 3.1 (positions 0–11557) belonging to the *enterolysin_A* class, and contig 14.6 (positions 4064–30979), which encodes *plantaricin_W beta*, classified as lanthipeptide Class II bacteriocins. These clusters encode antimicrobial peptides, such as hemolysin A, that inhibit competing bacterial strains by disrupting their membranes or interfering with vital cellular processes. Regulatory genes control bacteriocin expression in response to environmental signals, while transport-associated genes ensure efficient secretion and activation. Additionally, immunity-related genes protect *Lactobacillus* from self-toxicity by neutralizing its own bacteriocins. Collectively, these BGCs contribute to microbial competition, enhancing *Lactobacillus* survival, probiotic efficacy, and its role in modulating the gut microbiome. The BGCs organization was different than the reference strain WCFS1 (Figure 4B). Our previous genomic characterization of the *L. plantarum* UTNGt21A strain, isolated from *Solanum quitoense* (Lam.) fruits, revealed a similar overall genomic organization; however, the location of the bacteriocin-encoding genes differed (Tenea and Ascanta, 2022). The presence of enterolysin A in *Lactobacillus* UTNGt3, absent in the reference strain WCFS1, suggests a competitive advantage by enabling broader antimicrobial activity. As a class III bacteriocin, enterolysin A functions as a bacteriolysin, targeting bacterial cell walls and enhancing UTNGt3 ability to outcompete other microorganisms in diverse environments (Nilsen et al., 2003). This advantage may facilitate niche colonization, improve survival in competitive microbial ecosystems, and contribute to probiotic functions by suppressing pathogens and promoting gut health. The strain-specific presence of this bacteriocin could be the result of horizontal gene transfer or evolutionary adaptation, reflecting differences in ecological pressures between UTNGt3 and WCFS1. This genetic organization may account for the strong inhibitory activity of extracellular metabolites and precipitated peptides obtained from these native strains. Further analysis is currently underway to determine their chemical composition, which could provide deeper insights into their overall antimicrobial potency.

### 3.5 Genomic determinants of stress resilience, metabolic versatility, and host adaptation underpinning the probiotic potential of UTNGt3

A probiotic bacterium must be capable of surviving and temporarily persisting within the gastrointestinal tract, where it can exert beneficial effects on the host (Gizachew and Engidawork, 2024). However, a list of “probiotic marker genes” related to adhesion ability, bile salt hydrolase activity, gastrointestinal persistence, and resistance to different stressors (osmotic, oxidative, thermal, and acidic) were previously suggested in *L. plantarum* (Carpi et al., 2022; Garcia-Gonzalez et al., 2022). Their presence/absence and copy number is strain specific. From the gene annotation with Prokka and EggNOG several probiotic gene markers were detected in the UTNGt3 strain.

**FIGURE 5:**
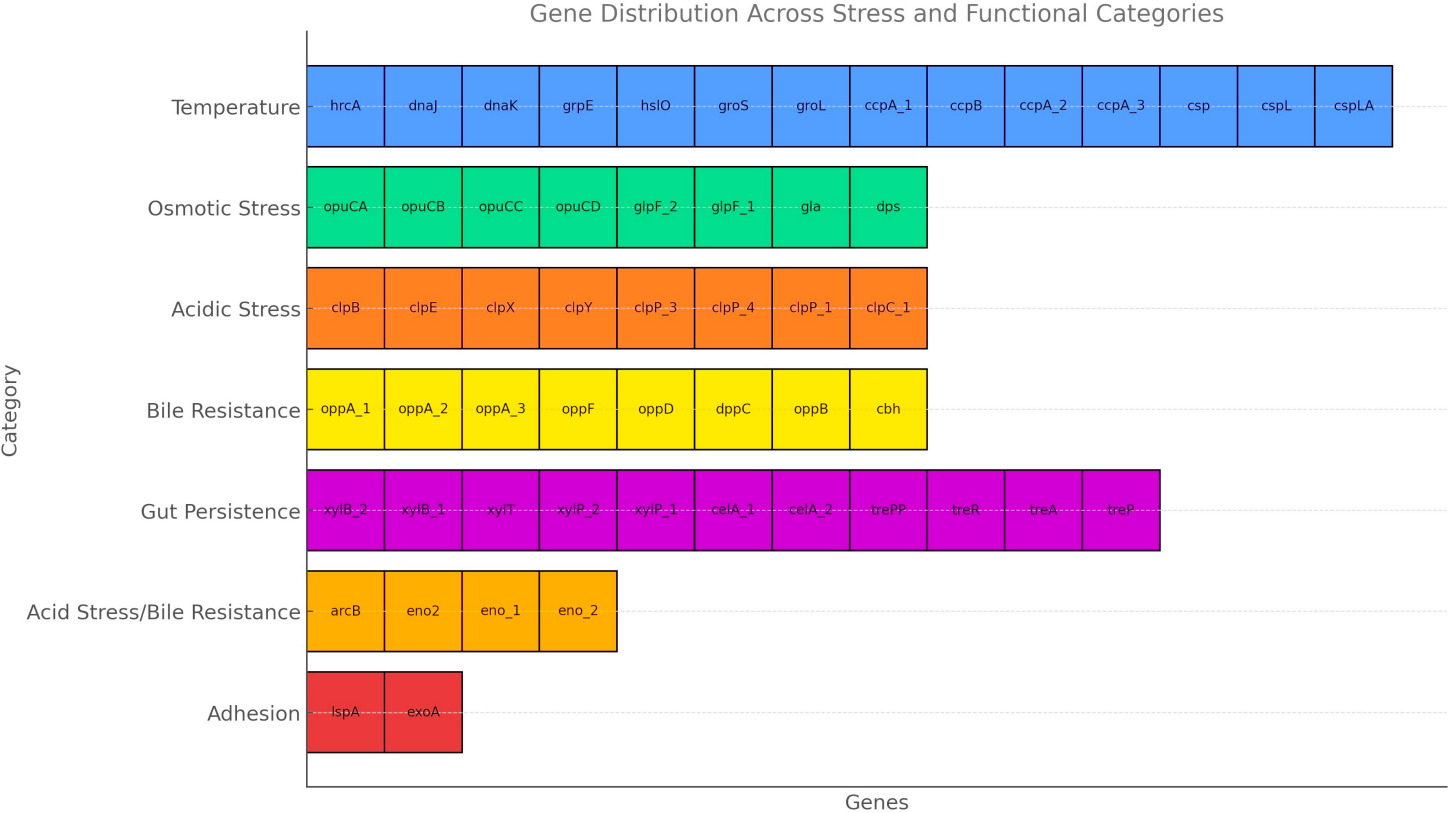
Probiotic marker genes and category distribution.

The gene distribution identified in the UTNGt3 strain highlights its strong probiotic potential, as it harbors a wide array of genes associated with stress tolerance and host interaction (Figure 5). Genes related to temperature and osmotic stress (e.g., *dna*K*, gro*L*, opu*CA*, glp*F) suggest resilience during food processing and storage, while those involved in acid and bile resistance (*clp*P*, clp*X*, cbh, opp*A) support survival through the harsh conditions of the gastrointestinal tract. Additionally, genes involved in carbohydrate metabolism and gut persistence (*xyl*P*, tre*PP*, cel*A*, cop*A*, cop*B*)* enhance its ability to adapt to the intestinal environment, and adhesion-related genes (*lsp*A*, exo*A) may facilitate colonization and interaction with the host epithelium. A detailed description of these probiotic markers is shown in Supplementary Table 7. In the study of Carpi et al. (2022), these probiotic gene markers varied with the strain reflecting the evolutionary adaptation of each *L. plantarum* strain to its unique ecological niche. For example, *cel*A gene (2 copies), *glp*F (2 copies) and *cop*B (1 copy) were annotated in the UTNGt3 but not the reference WCFS1. Even within the same species, genetic differences can lead to functional diversity, meaning one strain might excel in surviving acidic environments, while another might be better at adhering to intestinal cells or producing antimicrobial compounds. However, this variability underscores the importance of evaluating probiotic properties at the strain level rather than assuming all strains of a species behave the same way. In addition, strains from Amazonian fruits may show enhanced stress resilience due to exposure to natural microbial competition, fluctuating temperatures, and nutrient variability. These environments may facilitate horizontal gene transfer or promote the selective retention of advantageous genes, such as *treP* (involved in carbohydrate metabolism) and *clpX* (associated with stress response). Genome analysis of UTNGt3 predicts the presence of an extensive repertoire of metabolic and transport systems potentially supporting energy production, nutrient acquisition, and adaptation to gastrointestinal conditions. The genome encodes multiple phosphotransferase system (PTS) components, including oligo-mannoside-specific (*gmu*C), cellobiose-specific (*cel*A), chitobiose-specific (*chb*A), and trehalose-specific (*tre*A, *tre*P, *tre*R) transporters, suggesting capacity for utilization of a broad range of dietary and host-derived carbohydrates (Supplementary Table 7). Its metabolic flexibility is further increased by complementary non-PTS sugar transporters such isoprimeverose (*xyl*P) and D-xylose (*xyl*T) permeases. Genes encoding peptide uptake systems (*opp*A, *opp*B, *opp*C, *opp*D, *opp*F, and *dpp*C) may contribute to amino acid supply, growth, and potential bile tolerance under nutrient-variable conditions. Carnitine and glycine betaine transporters (*opu*CA–CD) contribute to osmoprotection, while glycerol facilitators (*glp*F, *gla*) increase energy metabolism under osmotic stress (Angelidis and Smith, 2003). Bile acid–modifying enzymes (*baiE*, *cbh*) suggest roles in bile detoxification and host–microbe interactions (Staley et al., 2017). Taken together, these genomic features imply metabolic versatility and ecological competitiveness in diverse niches, including the gastrointestinal tract. The presence of such genes likely contributes to the robustness of the isolate, enhancing its potential for industrial and functional food applications. Consequently, targeted screening for specific genetic markers can serve as a predictive tool for assessing a strain suitability for defined applications, including gut health promotion, stability in fermented products, and immune system modulation. Nonetheless, functional confirmation of these traits requires targeted *in vitro* and *in vivo* validation.

### 3.6 Riboflavin biosynthetic genes in UTNGt3 strain

**FIGURE 6:**
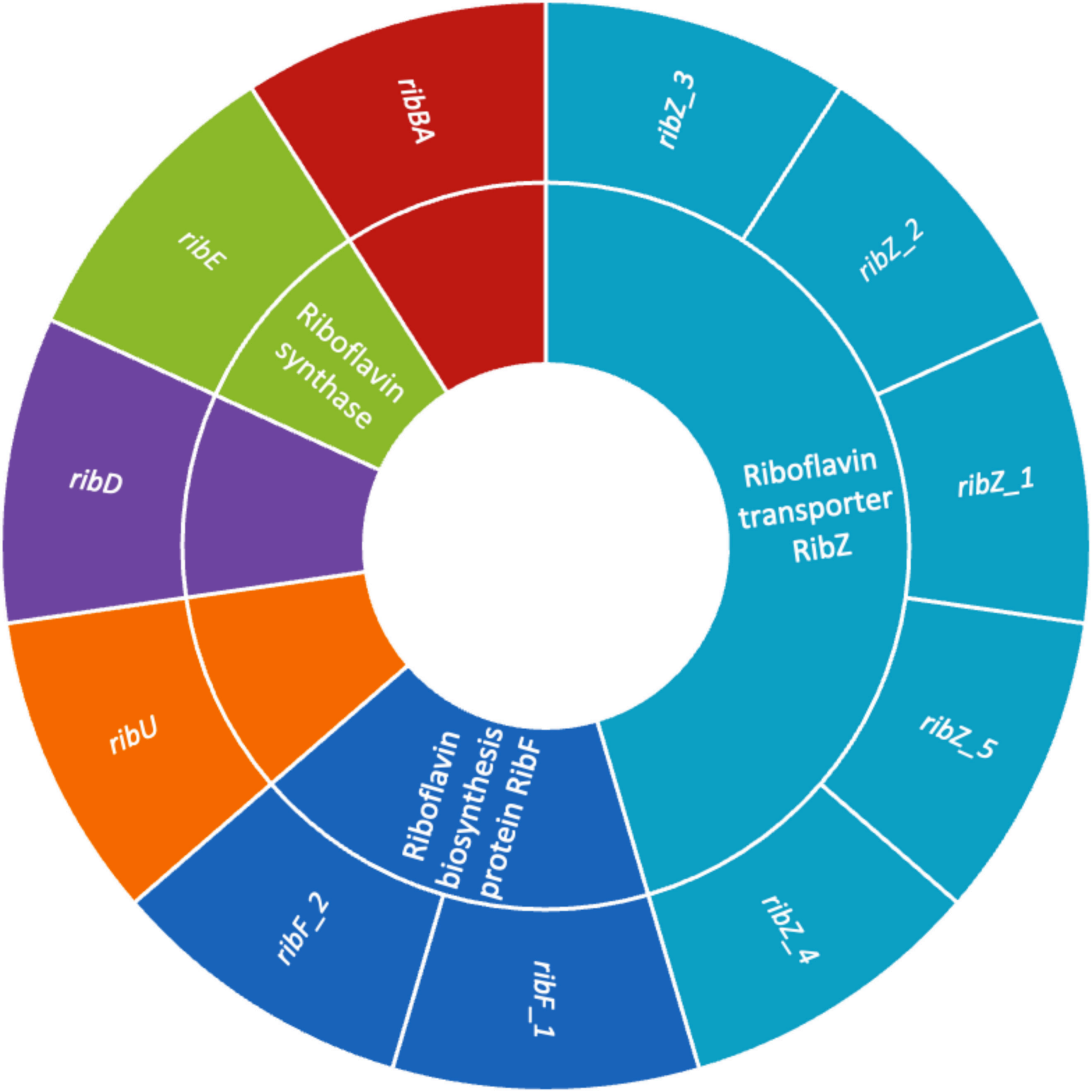
Sunburst chart showing the riboflavin biosynthesis and transport genes in UTNGt3. The inner ring represents the gene functions, and the outer ring listing associated gene variants.

The presence of genes encoding riboflavin (vitamin B2) biosynthesis in *Lactobacilli* is a significant trait that enhances their probiotic potential (Solopova et al., 2020). Figure 6 illustrates the presence of genes involved in riboflavin biosynthesis such as, *rib*AB, *rib*D, *rib*E, and *rib*F, which enable the bacterium to synthesize riboflavin *de novo*. This capability enhances the nutritional value of fermented foods (Averianova et al., 2020). Beyond nutritional enhancement, riboflavin plays a key role in stress resistance by supporting redox reactions through its coenzyme forms (FMN, FAD), helping probiotic strains withstand harsh gastrointestinal conditions like oxidative stress, low pH, and bile salts (Ashoori and Saedisomeolia, 2014). Additionally, riboflavin is involved in host immune modulation by influencing mucosal-associated invariant T (MAIT) cell activation, and it may help maintain gut barrier function (Germain et al., 2025). Riboflavin-producing *Lactobacilli* can also promote a healthier gut microbiome through cross-feeding. These features make riboflavin biosynthesis genes valuable markers for selecting robust probiotic strains suited for both industrial applications and targeted therapeutic uses (Averina et al., 2021). In addition, the presence of multiple riboflavin transporter genes, including *rib*Z_1, *rib*Z_2, *rib*Z_3, *rib*Z_4, *rib*Z_5 and *rib*U, highlights the strain enhanced ability to acquire riboflavin from its environment. Previous research indicate that the RibZ transporters are primarily responsible for facilitating riboflavin uptake under conditions where *de novo* synthesis might be limited, allowing the bacterium to maintain optimal intracellular levels of this essential vitamin (Gutiérrez-Preciado et al., 2015). Additionally, RibU, a high-affinity component of an ATP-binding cassette (ABC) transport system, ensures efficient riboflavin import, particularly under low-riboflavin conditions (Gutiérrez-Preciado et al., 2015). These transporter systems not only contribute to the bacterium’s metabolic flexibility and environmental adaptability but may also enhance its probiotic potential by improving riboflavin availability in the host or in fermented food products (Anumudu et al., 2024). The findings mark a significant advancement in creating fermented foods with higher riboflavin levels, produced *in situ*, eliminating the need for vitamin fortification. Such genetic profile reinforces strain value as a next-generation probiotic with intrinsic nutritional and health-promoting features.

### 3.7 *In vitro* probiotic attributes

#### 3.7.1. Tolerance to bile and synthetic gastric juice

**FIGURE 7:**
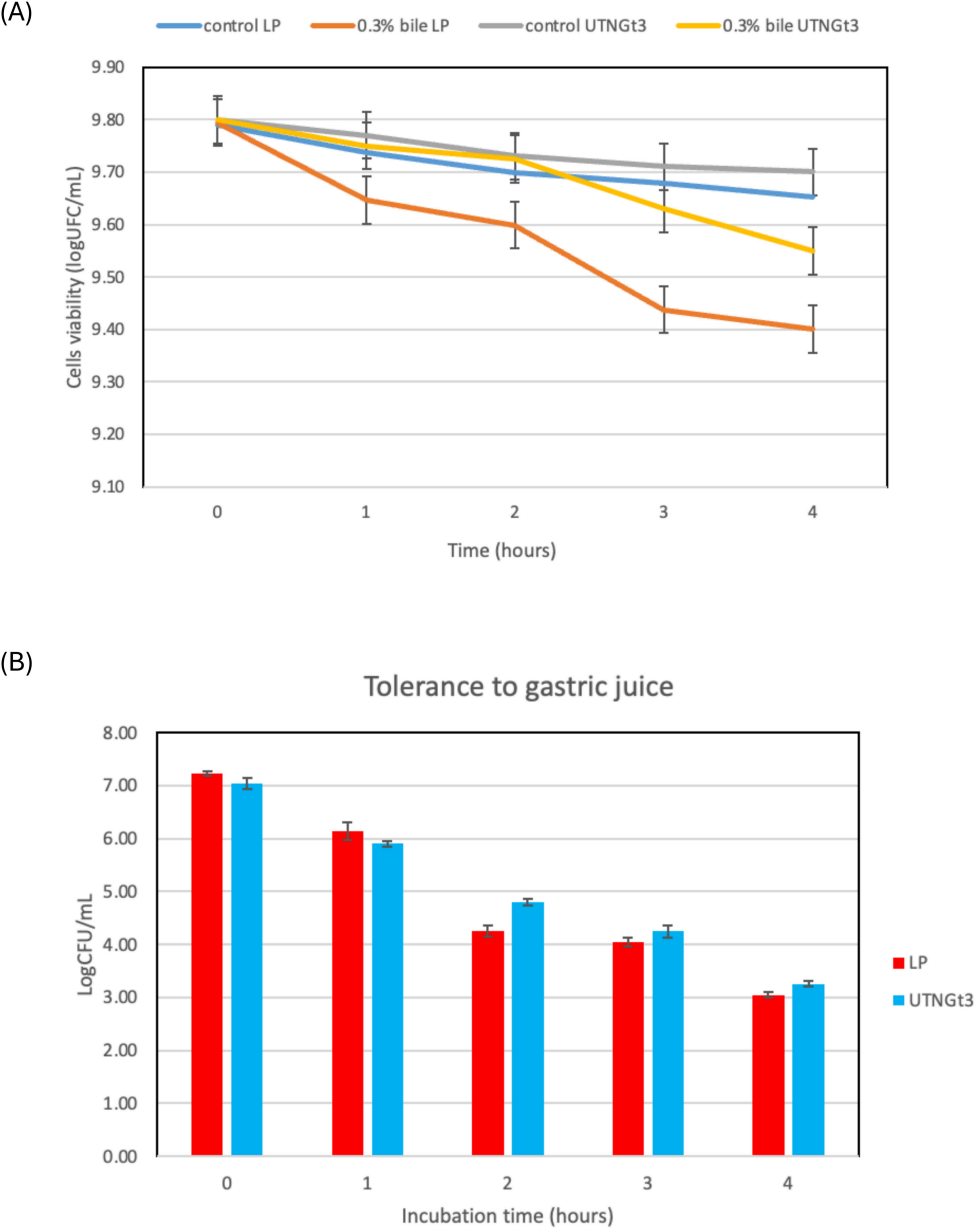
Cell viability (%) upon incubation with bile salt **(A)** and synthetic gastric juice **(B)** over time. Capital letters indicate the difference between conditions (LSD with Bonferroni correction); Small letters show the differences within the same strain (Duncan’s test). Legend: UTNGt3: *L. plantarum* UTNGt3; LP: *L. plantarum* ATCC8014.

Because of their function in regulating immune responses and fostering intestinal microbial balance, *Lactobacillus* strains have garnered more attention in recent years (Dempsey and Corr, 2022). The ability of a probiotic strain to survive stomach conditions and make it to the intestines undamaged is a basic requirement. The most important of the bactericidal substances found in gastric juice is hydrochloric acid, which lowers the pH of the stomach and makes it less conducive to microbial survival. This acidic environment poses a significant challenge for probiotics even though it is necessary for the removal of pathogens (Dempsey and Corr, 2022). Research suggests that a pH below 2.0 can be lethal to bacteria, though such extreme acidity is rarely maintained under normal conditions (Atasoy et al., 2024). This acidic environment poses an important obstacle for probiotics even though it is necessary for the removal of pathogens. Furthermore, the digestive enzyme pepsin, which breaks down proteins, intensifies bacterial destruction, making it even harder for beneficial microbes to enter the intestines (Sionek et al., 2024). To find strong probiotic strains that can survive digestion and successfully colonize the gut, it is essential to evaluate the effects under gastric simulation. However, in this study, the analysis of bile salts and gastric juice tolerance demonstrates that UTNGt3 exhibits superior resilience compared to LP, making it a stronger probiotic candidate. In bile tolerance assays, both strains start with similar viability (∼9.8 log CFU/mL), but while UTNGt3 maintains higher cell viability over time, LP experiences a steeper decline, particularly under 0.3% bile stress, indicating lower resistance (Figure 7A). Similarly, in gastric juice tolerance tests, UTNGt3 consistently survives better, with significantly higher CFU counts at each time point (Figure 7B). After 4 h, LP viability drops below 4 log CFU/mL, while UTNGt3 maintains a higher survival rate (∼5 log CFU/mL), suggesting enhanced acid resistance. The statistically significant differences (*p* < 0.05) confirm that UTNGt3 has superior resilience to harsh gastrointestinal conditions, allowing for greater survival during digestion and enhancing its potential for successful gut colonization. Similar studies on *L. plantarum* strain L15 isolated from a dairy product showed tolerance to different concentrations of bile salts as well as gastric acid demonstrated that this strain has a desirable potential for passing through the low pH of the stomach and entering the intestines (Alizadeh Behbahani et al., 2019). These results indicate that UTNGt3 has a better ability to withstand harsh gastrointestinal conditions, making it a more promising probiotic strain.

#### 3.7.2 Hydrophobicity, auto-aggregation, and adhesion to Caco-2 cells

**FIGURE 8:**
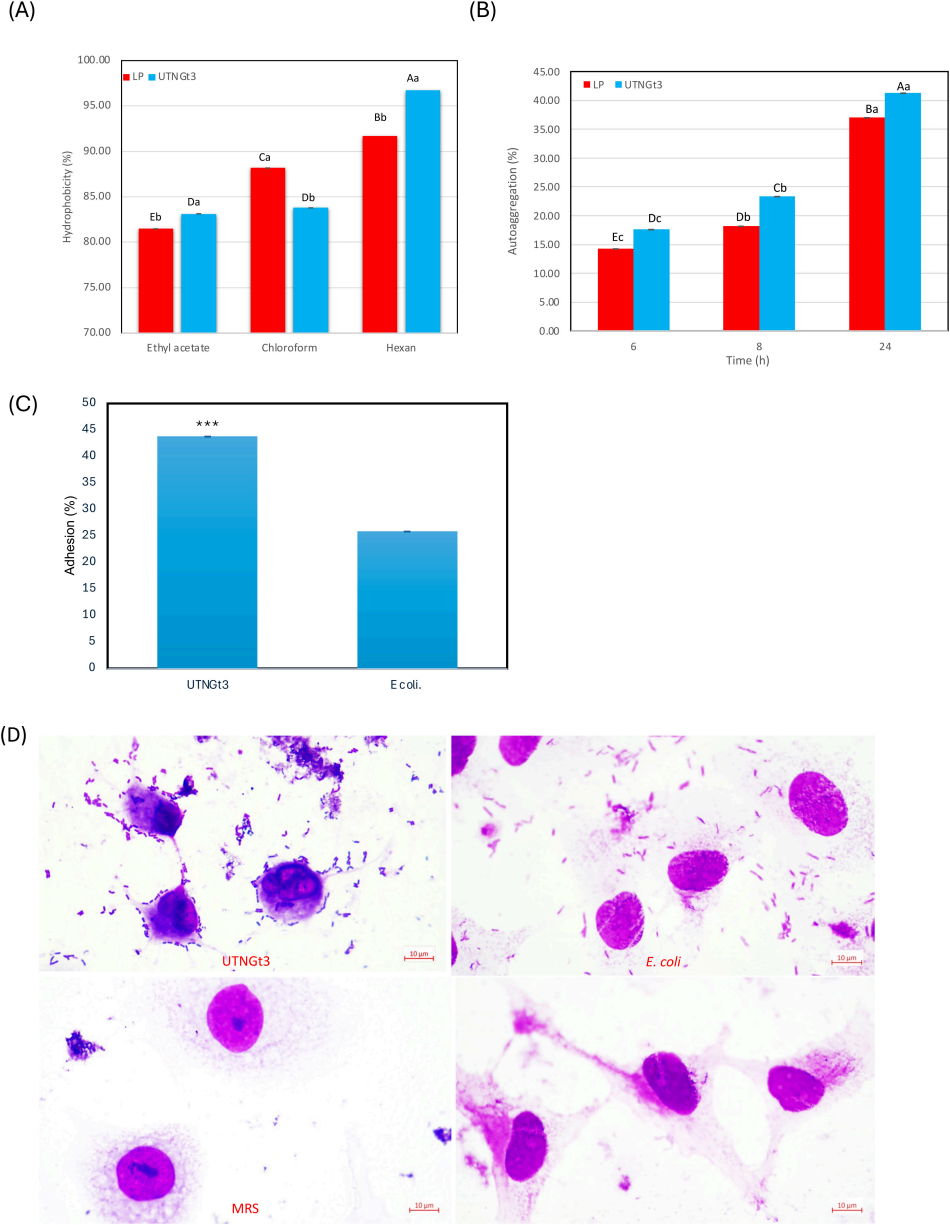
Graphical representation of the percentage of bacterial hydrophobicity **(A)** auto-aggregation **(B)**. Capital letters represent significant differences between strains (LSD with Bonferroni correction). Small letters represent differences within the same strain over time (Duncan’s test). Percentage of strains adherence to Caco-2 cells **(C)** and Representative microscopic observation (100 × ) **(D)**. The results represent the mean ± SD of three independent experiments. The significant difference was indicated by asterisks compared to *E. coli* ATCC ATCC11229: ***p < 0.05. Legend: UTNGt3: *L. plantarum* UTNGt3; LP: *L. plantarum* ATCC8014.

Hydrophobicity is a key physicochemical property of LAB that influences their ability to adhere to surfaces, including the intestinal epithelium and food matrices. High hydrophobicity enhances LAB attachment to intestinal epithelial cells, promoting gut colonization, competitive exclusion of pathogens, and immune modulation (Lau and Quek, 2024). It also aids in biofilm formation, contributing to natural food preservation and extended shelf life (Mgomi et al., 2023). Additionally, hydrophobicity improves LAB survival under stress conditions, such as acidic and osmotic challenges, enhancing their probiotic efficacy (Bustos et al., 2025). Some hydrophobic strains also produce bacteriocins, offering antimicrobial benefits (Mgomi et al., 2023). In this study, the hydrophobicity analysis of LP and UTNGt3 strains across three solvents (ethyl acetate, chloroform, and hexane) reveals statistically differences between the strains and solvents (Figure 8A). UTNGt3 exhibits slightly higher hydrophobicity in Ethyl Acetate (83.12%) and Hexane (96.73%), while LP shows greater hydrophobicity in chloroform (88.18%). The higher affinity of UTNGt3 for hexane suggests a more hydrophobic cell surface. Moreover, we observed that UTNGt3 showed similar autoaggregation pattern with LP (Figure 8B). Aggregation plays a crucial role in biofilm formation and is often associated with cell adherence properties, influencing bacterial survival and persistence in the gastrointestinal tract (Tuo et al., 2013). Besides, studies have shown a correlation between autoaggregation and adhesion ability in certain *LAB species* (Chantanawilas et al., 2024). Moreover, the results *showed that* UTNGt3 exhibits a strong adherence capacity to Caco-2 cells, suggesting a probiotic potential. The statistical analysis of adhesion reveals notable differences between the probiotic strain UTNGt3 and *E. coli* ATCC11229. The mean adhesion percentage for UTNGt3 was 43.74%, while *E. coli* ATCC11229 showed a lower adhesion rate of 25.84% (Figure 8C). The fold change calculation indicates that the probiotic strain adhered 1.69 times more effectively to Caco-2 cells than *E. coli*, suggesting a stronger ability to colonize. A *t*-test revealed a *p*-value of 0.0119, which is less than the 0.05 threshold for statistical significance. This confirms that the difference in adhesion between UTNGt3 and *E. coli* ATCC11229 is statistically significant. In conclusion, the probiotic strain UTNGt3 demonstrates significantly higher adhesion to Caco-2 cells, highlighting its potential for enhanced gut colonization and pathogen exclusion compared to *E. coli* ATCC11229. Figure 8D showed the attachment of the UTNGt3 and *E.coli* ATCC11229 to the Caco-2 cells under the microscope. UTNGt3 showed a localized and aggregative adherence, while *E.coli* showed diffuse adherence. Some Caco-2 cells exhibit morphological changes suggesting potential bacterial interaction effects. The lack of adherence in MRS and control samples confirms that the observed interactions are due to bacterial presence and not artifacts. Early study investigating the adherence of several lactobacilli to Caco-2 cells indicating that the percentage of adherence and their pattern is strain related (Wang et al., 2022). In another study, the adhesion potential of *L. plantarum* strain L15 to the human Caco-2 intestinal cell line, along with its auto- and co-aggregation abilities and anti-adherence activity against *E.coli* were evaluated (Alizadeh Behbahani et al., 2019). The strain demonstrated a capacity to survive stomach acidity and reach the intestines, with 54% hydrophobicity, 44% auto-aggregation, and 32% co-aggregation. Its adhesion to Caco-2 cells was 12%. Additionally, the strain exhibited significant anti-adherence effects, including competition, inhibition, and replacement against *E. coli*. These findings suggest that *L. plantarum* L15 has strong potential for antagonistic effects against *E. coli* (Alizadeh Behbahani et al., 2019).

### 3.8 Safety assessment of external metabolites from UTNGt3 on intestinal cells via MTT and LDH assays

**FIGURE 9:**
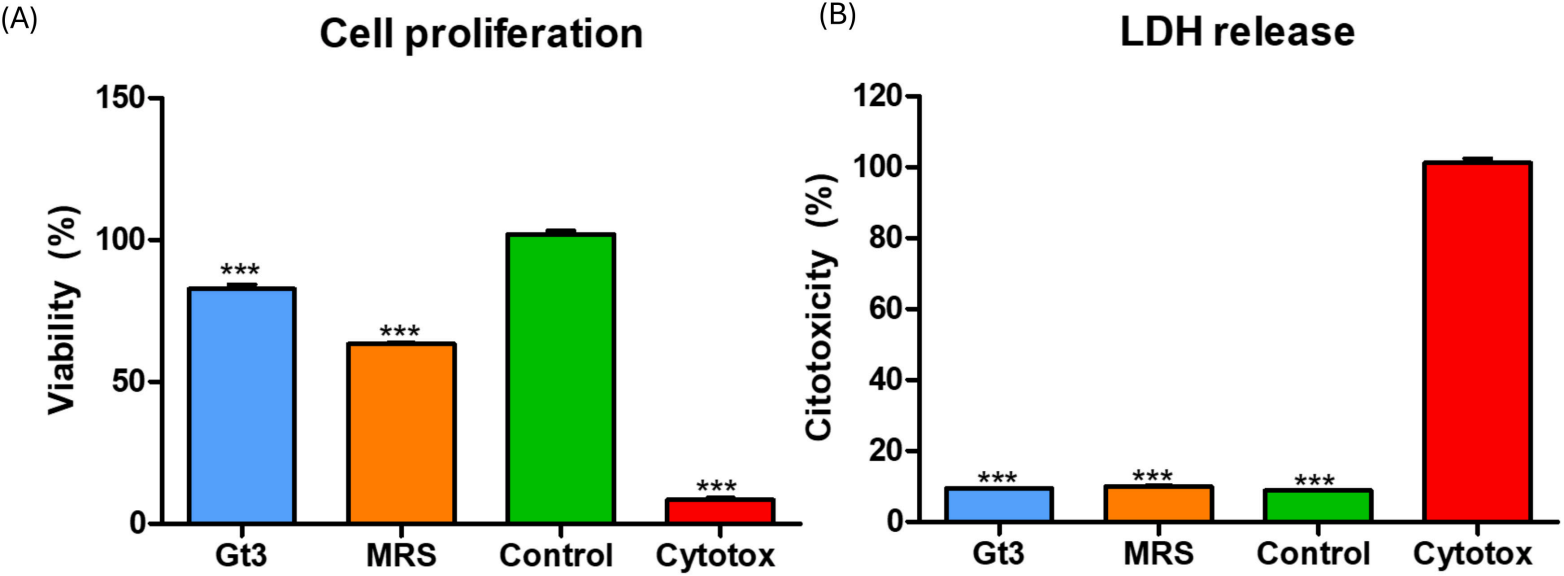
Effect of external metabolites from UTNGt3 on Caco-2 cells. **(A)** Cell viability was assessed using the MTT assay and expressed as a percentage relative to untreated control cells. The green bar represents the negative control (untreated cells), and the red bar represents the positive cytotoxic control (1% Triton X-100). Blue and light blue bars indicate strains with moderate to good biocompatibility, while the orange bar indicates reduced viability. **(B)** LDH assays. Results are expressed as the percentage of total LDH release relative to the positive control (1% Triton X-100, red bar). The green bar represents the negative control (untreated cells) and unstimulated cells in standard culture conditions (positive control). Data represent mean ± SEM. This condition was statistically significant compared to all other groups (***p < 0.001).

The MTT and LDH combined approach provided a robust evaluation of both mitochondrial activity and membrane integrity, allowing comprehensive assessment of external metabolites of UTNGt3 safety on intestinal epithelial cells (Figures 9A, B). The MTT assay results revealed that UTNGt3 preserved cell viability above 85%, indicating a non-cytotoxic effect and even potential supportive impact on cell proliferation. However, UTNGt3 showed slightly lower viability but still within a safe, non-toxic range (Figure 9A). In contrast, cells treated with MRS medium alone displayed a noticeable reduction in viability (∼60–70%), which suggests that the vehicle itself may exert a mild adverse effect in the absence of bacterial metabolites. As expected, the Triton X-100 (cytotoxic) group showed a drastic drop in viability (near 0%), confirming the assay sensitivity and serving as a robust positive control for cytotoxicity (Koley and Bard, 2010.). These results support the potential safety of Gt3 on colon epithelial cells, justifying their further exploration for probiotic or therapeutic use. The LDH cytotoxicity assay demonstrates that the culture supernatants of UTNGt3, do not induce significant membrane damage in host cells. The strain exhibited low LDH release levels, remaining below 10%, which is comparable to the negative (untreated) control (Figure 9B), indicated that CFS from UTNGt3 is non-cytotoxic and do not compromise cellular membrane integrity. The MRS medium, used as a vehicle control, induced a slightly higher LDH release than the bacterial supernatants but still within a non-toxic range, suggesting minor background effects of the medium itself. In contrast, the positive control (Triton X-100, labeled “Cytotox”) caused nearly 100% LDH release, confirming the sensitivity and reliability of the assay (Zipperer et al., 2023). This condition was statistically significant compared to all other groups (***, *p* < 0.001). Overall, these results, in agreement with the MTT proliferation assay, support the safety profile of the UTNGt3, indicating that they do not exert cytotoxic effects and are suitable for further investigation as potential probiotics or therapeutic agents.

The MTT assay results indicate that strain Gt3 preserves high Caco-2 cell viability, with values comparable to untreated controls, suggesting that its metabolites do not adversely affect mitochondrial function or overall metabolic activity. Concurrently, the LDH assay reveals minimal cytotoxicity, evidenced by low extracellular LDH levels, which reflect preserved membrane integrity and negligible cell lysis upon exposure to Gt3 culture supernatants. Collectively, these findings demonstrate the biocompatibility of Gt3 and support its safety for intestinal epithelial models. When benchmarked against other well-characterized probiotic strains, Gt3 exhibits an equivalent or superior cytocompatibility profile. For example, *Lactobacillus plantarum* PBS067 and *L. rhamnosus* PBS070 have been reported to maintain high viability and low cytotoxicity in HT-29 cells, a commonly used epithelial model (Presti et al., 2015). Similarly, postbiotic preparations from *L. fermentum* showed no significant LDH release in Caco-2 cells, suggesting compatibility with the intestinal barrier (Dinić et al., 2017). Moreover, *L. acidophilus* LA-5 demonstrated low cytotoxicity in human peripheral monocytes, with LDH levels indistinguishable from controls (Vale et al., 2023). These comparisons reinforce the safety and potential of Gt3 for probiotic or postbiotic applications.

A recent study evaluated the wound healing potential of *L. reuteri* NCHBL-005, a probiotic strain isolated from honeybees, using L929 fibroblasts and mouse embryonic fibroblasts as model systems. The findings demonstrated that *L. reuteri* NCHBL-005 significantly enhanced fibroblast-mediated wound closure both *in vitro* and *in vivo*. Enhanced cellular proliferation was confirmed via MTT assay, with the most pronounced effect observed 72 h post-treatment, suggesting a stimulatory effect on fibroblast growth and tissue regeneration (Kim et al., 2025). In a separate investigation, *L. gasseri* SF1183 was shown to modulate intestinal epithelial cell proliferation. Conditioned media from this strain reversibly reduced the proliferation of HCT116 colorectal cancer cells, indicating a regulatory effect on epithelial cell turnover, potentially contributing to gut homeostasis (Di Luccia et al., 2022). Additionally, several *Lactobacillus* strains have been reported to exert selective cytotoxic effects on cancer cell lines via mechanisms involving apoptotic induction and membrane disruption. For instance, Chen et al. (2017) demonstrated that strain PM177 significantly decreased HT-29 cell viability (IC50 = 134.9 μL/mL) as assessed by MTT assay, while BCRC14625 induced substantial LDH release, indicative of compromised membrane integrity. These findings collectively underscore the diverse functional capacities of *Lactobacillus* strains in modulating cell proliferation, promoting tissue repair, and exerting anti-cancer effects.

## 4 Conclusion

*L. plantarum* UTNGt3 exhibits a unique combination of genomic, functional, and phenotypic features that position it as a strong probiotic candidate for functional food and gut health applications. The strain relatively large genome, enriched with coding sequences and prophage-associated elements, suggests enhanced genetic plasticity, stress resilience, and ecological adaptability. While intrinsic antibiotic resistance genes were detected, the lack of mobile genetic elements or evidence of horizontal gene transfer minimizes associated safety concerns, aligning with EFSA probiotic safety criteria. Furthermore, the presence of multiple BGCs, including those encoding plantaricin variants, underpins UTNGt3 potent antimicrobial activity, which likely confers a competitive advantage within complex microbial ecosystems. The strain harbors a broad repertoire of probiotic marker genes implicated in acid and bile tolerance, thermal resilience, adhesion, and host-microbe interaction, supporting its functional adaptation to gastrointestinal transit and colonization. Phenotypic validation confirmed UTNGt3 strong tolerance to gastric and bile conditions, robust adhesion to intestinal epithelial cells, and its capacity for riboflavin biosynthesis, a valuable trait for functional food fortification. Additionally, *in vitro* cytotoxicity assays, demonstrated that extracellular metabolites from UTNGt3 maintain over 85% cell viability and membrane integrity in Caco-2 cells, highlighting its safety profile for oral consumption. Collectively, these findings validate UTNGt3 as a promising multifunctional probiotic strain suitable for integration into food matrices or therapeutic formulations. Future research should focus on *in vivo* validation of UTNGt3 probiotic efficacy and safety using animal models. Additionally, its antimicrobial spectrum against enteric pathogens warrants further investigation, along with the stability and functional performance of the strain in different food delivery systems. Metabolomic and transcriptomic profiling under gastrointestinal conditions will also help elucidate its adaptive mechanisms and interaction with host microbiota, paving the way for its application in precision probiotics and personalized nutrition strategies.

## Data Availability

The datasets presented in this study can be found in online repositories. The names of the repository/repositories and accession number(s) can be found in the article/Supplementary material.

## Footnotes

1
http://gctype.wdcm.org

2
https://metacyc.org

3
https://www.ncbi.nlm.nih.gov/sra/PRJNA1116628
