# Supplementary material for: Genomic and functional insights into Lactiplantibacillus plantarum UTNGt3 from Ecuadorian Amazon Chrysophyllum oliviforme: a safe and promising probiotic
Source: Front Microbiol. 2025 Sep 10;16:1634475. doi: 10.3389/fmicb.2025.1634475 (PMC12457330; doi:10.3389/fmicb.2025.1634475)
Supplement: Supplementary file 1 [file Table_1.docx]

**Supplementary files**

**Genomic and Functional Insights into Lactiplantibacillus plantarum UTNGt3 from Ecuadorian Amazon Chrysophyllum oliviforme: A Safe and Promising Probiotic**

Gabriela N. Tenea^1*^, Jazmin Hidalgo^1^, Gratiela Gradisteanu Pircalabioru^2,3^, Victor Cifuentes^1^

^1^Biofood and Nutraceutics Research and Development Group; Faculty of Engineering in Agricultural and Environmental Sciences, Universidad Técnica del Norte, Ibarra, Ecuador.

^2^ Research Institute of the University of Bucharest—ICUB, University of Bucharest, 91-95 Splaiul Independentei St., 5050095 Bucharest, Romania

^3^ eBio-hub Centre of Excellence in Bioengineering, National University of Science and Technology Politehnica Bucharest

* Corresponding author: gntenea@utn.edu.ec

**Table S1.** Assembly summary of contigs

| Sample | Contigs | Total contig bases | N50 | Max length | Min length | Mean length |
| --- | --- | --- | --- | --- | --- | --- |
| UTNGt3 | 37 | 3,569,352 | 245,269 | 575,670 | 4163 | 96,468 |

Contigs: The number of contigs assembled

Total bases of contigs : The total length of contigs

N50: 50% of all contig bases come from contigs longer than this value

Max length: The length of the longest contig

Min length: The length of the shortest contig

Mean length: The average length of contigs assembled

**Table S2.** Mapping overall results

| Library name | Total reads | Mapped reads | Coverage (%) | Depth | Ins.size (Std.) |
| --- | --- | --- | --- | --- | --- |
| UTNGt3 | 17,301,560 | 17,283,962 (99.90%) | 100.00 | 697,65 | 422,06 (98.75) |

Library name : Sample’s library name

Total reads : Total number of reads

Mapped reads : Total number of mapped reads

Coverage (%) : The percentage of mapped sited (>= 1x)

Depth : Average mapping depth

Ins.size (Std.) : The length between adapters and standard deviation of predicted length

**Table S3.** Busco analysis result

*Used Lineage : bacteria_odb10 (number of genomes: 4085, number of BUSCOs: 124)*

| Status | # of BUSCOs | Percentage (%) |
| --- | --- | --- |
| Complete BUSCOs (C) |  | |
| Complete and single-copy BUSCOs (S) | 123 | 99.19 |
| Complete and duplicated BUSCOs (D) | 1 | 0.81 |
| Fragmented BUSCOs (F) | 0 | 0.00 |
| Missing BUSCOs (M) | 0 | 0.00 |
| Total BUSCO groups searched | 124 | 100 |

Status : A quantitative assessment list of the completeness in terms of expected gene content.

The following two conditions are used to create a status:

a. Expected range of scores

b. Expected range of length alignments

If both conditions are met, it is classified as Complete (These complete busco matches are

either single-copy or duplicated). If length alignments is not met, it is classified as Fragmented.

If both conditions are not met, it is classified as Missing.

# of BUSCOs : Identified count in sample

Percentage : Identified percentage in sample

**Table S4.** List of genomes used for typing

| **Accession No.** | **Description** |
| --- | --- |
| GCA_018982875.1 | *Lactiplantibacillus pentosus O17* |
| GCA_016804305.1 | *Lactiplantibacillus pentosus MS031* |
| GCA_003702565.1 | *Lactiplantibacillus pentosus IG12* |
| GCA_900092635.1 | *Lactiplantibacillus pentosus MP-10* |
| GCA_003641185.1 | *Lactiplantibacillus pentosus DSM20314* |
| GCA_002850015.1 | *Lactobacillus pentosus BGM48* |
| GCA_018403455.1 | *Lactobacillus pentosus AWA1501* |
| GCA_004354685.1 | *Lactiplantibacillus pentosus ATCC 8041* |
| GCA_003627375.1 | *Lactiplantibacillus pentosus ZFM94* |
| GCA_001434175.1 | *Lactobacillus plantarum subsp. plantarum ATCC 14917 = JCM 1149 = CGMCC 1.2437* |
| GCF_000203855.3 | *Lactobacillus plantarum WCFS1* |
| GCA_016406265.1 | *Lactococcus lactis WiKim0098* |
| GCA_002158885.1 | *Lactobacillus gasseri 4M13* |
| GCA_000014465.1 | *Lactobacillus brevis ATCC 367* |
| GCA_002370355.1 | *Lactobacillus sakei subsp. sakei DSM 20017 = JCM 1157* |
| GCA_002849915.1 | *Lactobacillus helveticus FAM8627* |
| GCA_000466785.3 | *Lactobacillus fermentum 3872* |
| GCA_001438695.1 | *Fructobacillus fructosus KCTC 3544* |
| GCA_018363095.1 | *Lacticaseibacillus casei FBL6* |
| GCA_002055965.1 | *Bacillus subtilis NCIB 3610* |
| GCA_016653515.1 | *Lacticaseibacillus rhamnosus KF7* |
| GCA_001027105.1 | *Staphylococcus aureus DSM20231* |

**Table S5.** Distribution of CAZyme families identified in *L. plantarum* UTNGt3 genome based on genome annotations.

| **CAZyme Class** | **Family Codes** | **Number of Genes** |
| --- | --- | --- |
| Glycoside Hydrolases (GH) | GH1, GH2, GH8, GH13, GH20, GH23, GH25, GH32, GH36, GH38, GH42, GH65, GH73, GH170, GH0 | 10, 2, 1, 10, 1, 2, 7, 2, 2, 4, 2, 4, 3, 4, 4 |
| Glycosyltransferases (GT) | GT2, GT4, GT5, GT26, GT28, GT30, GT51, GT111 | 19, 15, 1, 2, 1, 1, 2, 1 |
| Carbohydrate-Binding Modules (CBM) | CBM0, CBM32, CBM50 | 1, 1, 12 |
| Carbohydrate Esterases (CE) | CE9, CE12 | 1, 1 |
| Auxiliary Activities (AA) | AA10 | 1 |

**Table S6.** Virulence genes predicted in the UTNGt3 genome with VFDB database.

| **Gene** | **Similarity (%)** | **Description** |
| --- | --- | --- |
| gene01673 | 75.7 | VFG005871(gb\|WP_002991444) (*has*C) UTP--glucose-1-phosphate uridylyltransferase HasC |
| gene01696 | 70.4 | VFG000077(gb\|NP_465991) (*clp*P) ATP-dependent Clp protease proteolytic subunit |
| gene01702 | 72 | VFG005582(gb\|WP_002897814) (*eno*) phosphopyruvate hydratase |
| gene01769 | 76.8 | VFG006826(gb\|NP_464902) (*lis*R) two-component response regulator |
| gene02317 | 79.6 | VFG006022(gb\|WP_002947383) (*rfb*B) dTDP-glucose 4,6-dehydratase |
| gene02319 | 77 | VFG005898(gb\|WP_002904719) (*rfb*A) glucose-1-phosphate thymidylyltransferase RfbA |
| gene02475 | 71.2 | VFG046474(gb\|WP_014714676) (*tuf*A) elongation factor Tu |

**Table S7.** Stressors/ Probiotic marker genes list annotated in the UTNGt3 genome

| **Category** | **Gene** | **Description** |
| --- | --- | --- |
| **Temperature** | *hrcA* | Heat-inducible transcription repressor HrcA |
|  | *dnaJ* | Chaperone protein DnaJ |
|  | *dnaK* | Chaperone protein DnaK |
|  | *grpE* | Protein GrpE |
|  | *hslO* | 33 kDa chaperonin |
|  | *groS* | 10 kDa chaperonin |
|  | *groL* | 60 kDa chaperonin |
|  | *ccpA_1* | Catabolite control protein A |
|  | *ccpB* | Catabolite control protein B |
|  | *ccpA_2* | Catabolite control protein A |
|  | *ccpA_3* | Catabolite control protein A |
|  | *csp* | Cold shock protein 1 |
|  | *cspL* | Cold shock protein 2 |
|  | *cspLA* | Cold shock-like protein CspLA |
| Osmotic stress | *opuCA* | Carnitine transport ATP-binding protein OpuCA |
|  | *opuCB* | Carnitine transport permease protein OpuCB |
|  | *opuCC* | Glycine betaine/carnitine/choline-binding protein OpuCC |
|  | *opuCD* | Carnitine transport permease protein OpuCD |
|  | *glpF_2* | Glycerol uptake facilitator protein |
|  | *glpF_1* | Glycerol uptake facilitator protein |
|  | *gla* | Glycerol facilitator-aquaporin gla |
|  | *dps* | DNA protection during starvation protein |
| Acid stress | *argS* | Arginine--tRNA ligase |
|  | *atpB* | ATP synthase subunit a |
|  | *atpE* | ATP synthase subunit c |
|  | *atpF* | ATP synthase subunit b |
|  | *atpH* | ATP synthase subunit delta |
|  | *atpA* | ATP synthase subunit alpha |
|  | *atpG* | ATP synthase gamma chain |
|  | *atpD* | ATP synthase subunit beta |
|  | *atpC* | ATP synthase epsilon chain |
|  | *clpB* | Chaperone protein ClpB |
|  | *clpE* | ATP-dependent Clp protease ATP-binding subunit ClpE |
|  | *clpX* | ATP-dependent Clp protease ATP-binding subunit ClpX |
|  | *clpY* | ATP-dependent protease ATPase subunit ClpY |
|  | *clpP_3* | ATP-dependent Clp protease proteolytic subunit |
|  | *clpP_4* | ATP-dependent Clp protease proteolytic subunit |
|  | *clpP_1* | ATP-dependent Clp protease proteolytic subunit |
|  | *clpC_1* | putative ATP-dependent Clp protease ATP-binding subunit |
|  | *copA_1* | putative copper-importing P-type ATPase A |
|  | *copA_2* | Copper-exporting P-type ATPase |
|  | *copB* | Copper-exporting P-type ATPase B |
|  | *gadB* | Glutamate decarboxylase |
|  | *gap* | Glyceraldehyde-3-phosphate dehydrogenase |
|  | *relA* | GTP pyrophosphokinase |
|  | *recA* | Protein RecA |
|  | *tpiA* | Triosephosphate isomerase |
| Bile resistance | *oppA_1* | Oligopeptide-binding protein OppA |
|  | *oppA_2* | Oligopeptide-binding protein OppA |
|  | *oppA_3* | Oligopeptide-binding protein OppA |
|  | *oppF* | Oligopeptide transport ATP-binding protein OppF |
|  | *oppD* | Oligopeptide transport ATP-binding protein OppD |
|  | *dppC* | Dipeptide transport system permease protein DppC |
|  | *oppB* | Oligopeptide transport system permease protein OppB |
|  | *cbh* | Choloylglycine hydrolase |
|  | *pdhA* | Pyruvate dehydrogenase E1 component subunit alpha |
|  | *pdhB* | Pyruvate dehydrogenase E1 component subunit beta |
|  | *pdhC* | Dihydrolipoyllysine-residue acetyltransferase component of pyruvate dehydrogenase complex |
|  | *pdhD_1* | Dihydrolipoyl dehydrogenase |
|  | *pdhD_2* | Dihydrolipoyl dehydrogenase |
|  | *nagB* | Glucosamine-6-phosphate deaminase |
| Gut persistence/ gut metabolism | *xylB_2* | Xylulose kinase |
|  | *xylB_1* | Aryl-alcohol dehydrogenase |
|  | *xylT* | D-xylose transporter |
|  | *xylP_2* | Isoprimeverose transporter |
|  | *xylP_1* | Isoprimeverose transporter |
|  | *celA_1* | PTS system cellobiose-specific EIIB component |
|  | *celA_2* | PTS system cellobiose-specific EIIB component |
|  | *gmuC_3* | PTS system oligo-beta-mannoside-specific EIIC component |
|  | *chbA* | PTS system N,N'-diacetylchitobiose-specific EIIA component |
|  | *trePP* | Trehalose 6-phosphate phosphorylase |
|  | *treR* | HTH-type transcriptional regulator TreR |
|  | *treA* | Trehalose-6-phosphate hydrolase |
|  | *treP* | Alpha,alpha-trehalose phosphorylase |
|  | *baiE* | Bile acid 7-alpha dehydratase |
| Acid stress/ bile resistance | *arcB* | Ornithine carbamoyltransferase, catabolic |
|  | *eno2* | Enolase 2 |
|  | *eno_1* | Enolase |
|  | *eno_2* | Enolase |
| Adhesion | *lspA* | Lipoprotein signal peptidase |
|  | *exoA* | Exodeoxyribonuclease |
| Oxidative stress | *msrA_1* | Peptide methionine sulfoxide reductase MsrA |
|  | *msrA_2* | Peptide methionine sulfoxide reductase MsrA |
|  | *msrB* | Peptide methionine sulfoxide reductase MsrB |
| Adhesion/ Bile resistance | *UTNGt3_00500* | sortase family |

**Figure S1**. COG (A) and KEGG (B) gene annotation. Gene number and category are shown.

(A)


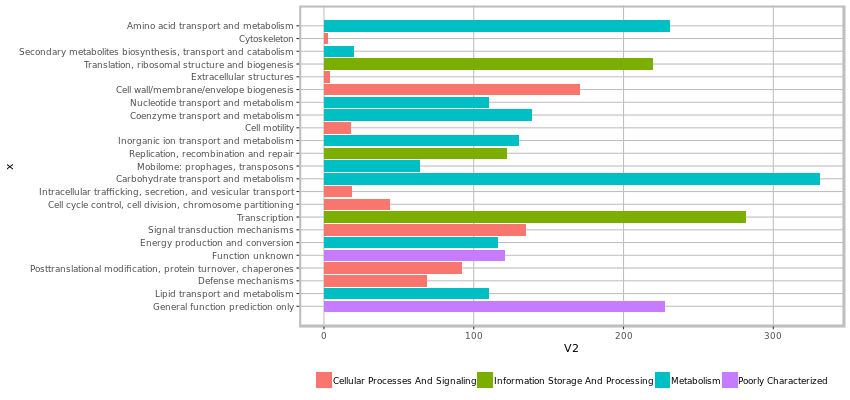


(B)


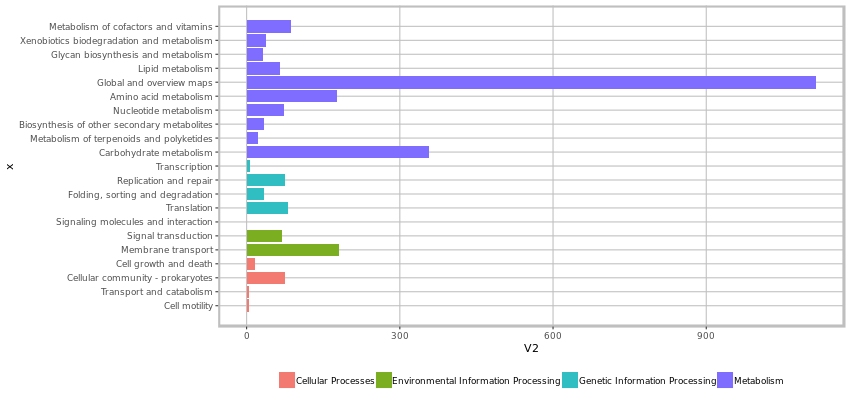


**Figure S2.** ANI analysis. Percent identity heatmap resulted (A). Alignment coverage heatmap resulted (B). The cells in the heatmap corresponding to an ANI value of 95% and higher are stained red. This indicates that the corresponding strains belong to the same species. The dendrograms (in green; above and on the left side), which were constructed by the simple linkage of the ANIm (ANI with MUMmer) percentage identities, correspond to the results of the clustering of the ANI values between the used strains (Pritchard et al., 2016). The isolates and species assignments as indicated at source are given as row and column labels. Cells in the heatmap corresponding to 75% coverage or greater are colored red. Color intensity fades as the comparisons approach 50% coverage. The dendrograms (in green; above and on the left side) of the heatmap correspond to strains assignments for each isolate in the analysis. ANI: Average Nucleotide Identity.
